# Single-cell isoform analysis in human immune cells

**DOI:** 10.1186/s13059-022-02615-z

**Published:** 2022-02-07

**Authors:** Roger Volden, Christopher Vollmers

**Affiliations:** Department of Biomolecular Engineering, University of California Santa Cruz, Santa Cruz, CA 95064 USA

## Abstract

High-throughput single-cell analysis today is facilitated by protocols like the 10X Genomics platform or Drop-Seq which generate cDNA pools in which the origin of a transcript is encoded at its 5′ or 3′ end. Here, we used R2C2 to sequence and demultiplex 12 million full-length cDNA molecules generated by the 10X Genomics platform from ~3000 peripheral blood mononuclear cells. We use these reads, independent from Illumina data, to identify B cell, T cell, and monocyte clusters and generate isoform-level transcriptomes for cells and cell types. Finally, we extract paired adaptive immune receptor sequences unique to each T and B cell.

**Supplementary Information:**

The online version contains supplementary material available at 10.1186/s13059-022-02615-z.

## Introduction

The analysis of transcriptomes using high-throughput sequencers has revolutionized biomedical research [1, 2]. Pairing transcriptome analysis with the high-throughput processing of single cells has provided unprecedented insight into cellular heterogeneity [3, 4]. Among many other studies, researchers have leveraged the strengths of high-throughput single-cell transcriptome analysis to create single-cell maps of the mouse [5, 6] or *C*. *elegans* [7] model organisms, to elucidate a new cell type in the lung involved in cystic fibrosis [8], and to increase our knowledge of adaptive and innate immune cells [9–12].

High-throughput single-cell transcriptome analysis however comes with trade-offs. In particular, droplet- or microwell-based methods like Drop-seq [13], InDrop, 10X Genomics [14], and Microwell-Seq [6] or Seq-Well [15] single-cell workflows generate pools of full-length cDNA with either the 5′ or 3′ end containing cellular identifiers. The cDNA pools are intended for high-throughput short-read sequencing and must therefore be fragmented such that one read sequence includes the cellular identifier and the sequence of its pair includes a fragment from within the original cDNA molecule. As a result, only a relatively short fragment of the cDNA is then sequenced alongside the cellular identifier limiting the resolution of this approach to the identification of genes associated with a given molecular identifier.

Instead of sequencing transcript fragments, long-read sequencing methods in the form of Pacific Biosciences (PacBio) and Oxford Nanopore Technologies (ONT) are now capable of sequencing comprehensive full-length transcriptomes [16–19]. These methods have now been used to analyze single-cell cDNA pools generated by different methods, both well- [20–22] and droplet-based [23–27], enriching the information we can extract from single-cell experiments. However, for the analysis of high-throughput droplet-based experiments with long reads, short-read data are still required for interpreting experimental data [27, 28] or enabling the identification of cellular and molecular identifiers in low-accuracy ONT reads [27]. Short-read data remain a requirement because either long-read data are not of sufficient depth to cluster cells into cell types or not accurate enough to decode the cellular origin of cDNA molecules.

Because decoding the cellular origin of a cDNA molecule requires accurate sequencing of the molecular identifier, error-prone long read technologies are generally not sufficient to sequence each cDNA pool and to accurately interpret the single-cell data encoded therein. We have previously developed and applied the R2C2 approach which uses concatemeric consensus sequencing to improve ONT read accuracy from ~92 to >99% while still producing more than 2 million full-length cDNA sequences per MinION flow cell [19, 20, 29, 30]. This increase in accuracy however comes with a decrease in read throughput as regular cDNA ONT runs can yield from 10 to 20 million reads per MinION flow cell.

In this manuscript, we demonstrate that this combination of high throughput and accuracy of the R2C2 method is sufficient for the Illumina short-read independent analysis of highly multiplexed single-cell cDNA pools generated by the 10x Genomics Chromium controller. We independently analyzed two pools containing the cDNA molecules with a combined ~3000 human *p*eripheral *b*lood *m*ononuclear *c*ells (PBMCs) with Illumina and the established R2C2 [20] (ONT) workflows. To this end, we modified the R2C2 workflow to be compatible with cDNA generated by the 10x Chromium controller and implemented new computational tools to identify 10x molecular and cellular identifiers. By merging reads based on the molecular identifiers and demultiplexing reads based on their cellular identifiers, we showed that the R2C2 approach identifies the same cellular identifiers in the cDNA pools and generates comparable single-cell gene expression profiles and cell type clusters as Illumina-based sequencing. In addition, and in contrast to Illumina data, R2C2 data also allow the determination of cell type-specific and single-cell isoform-level transcriptomes. Finally, we developed a set of computational tools that allowed us to process R2C2 data to resolve and pair full-length adaptive immune receptor (AIR) transcripts in the B and T cell subpopulations of our PBMC sample which currently requires specialized library preparation methods and sequencing approaches.

## Results

**Fig. 1:**
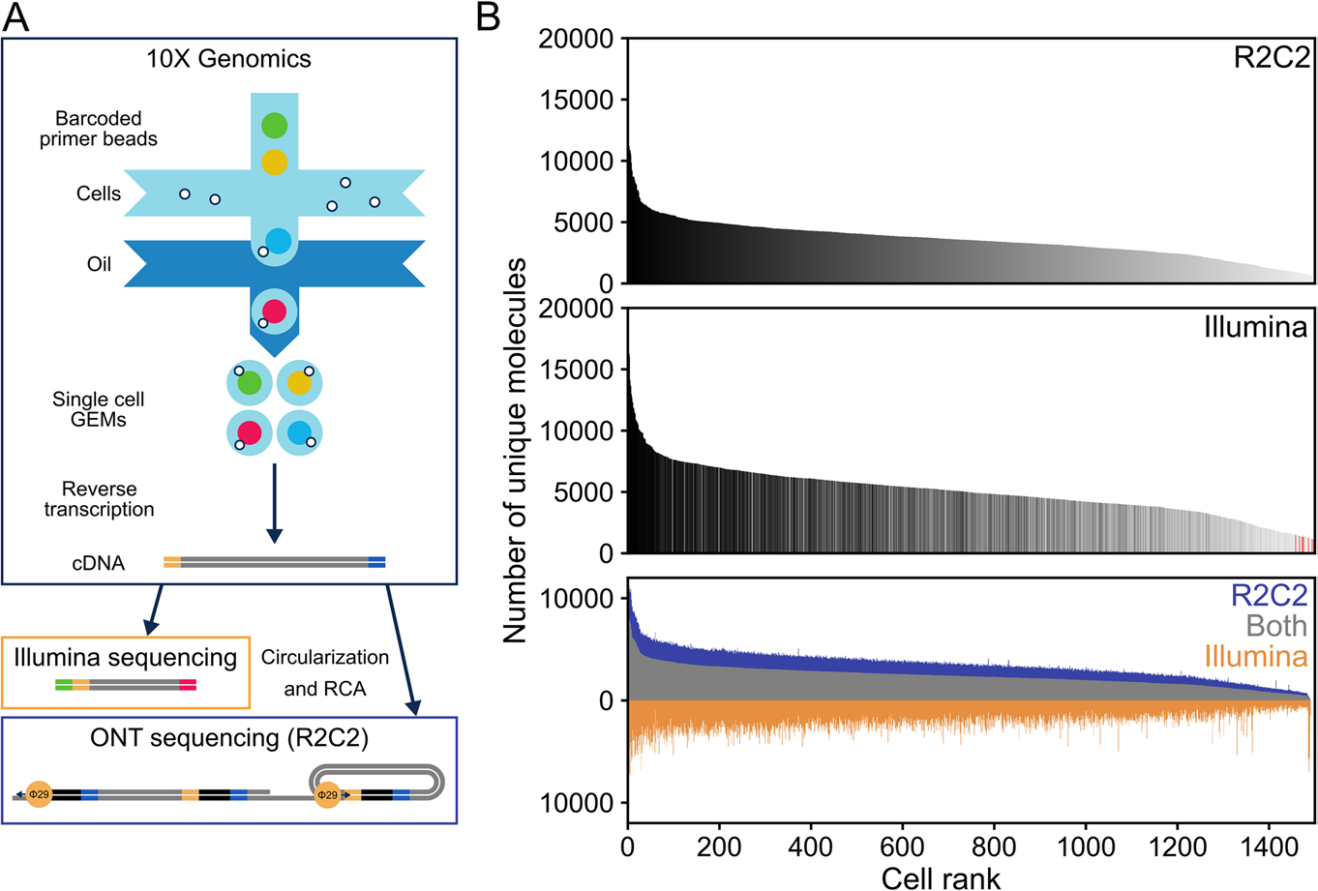
Data generation and characteristics. **A** Thousands of peripheral blood mononuclear cells (PBMCs) were processed using the 10X Genomics Chromium Single Cell 3′ Gene Expression Solution. The resulting full-length cDNA was either fragmented for Illumina sequencing or processed using the R2C2 workflow. **B** After read processing and demultiplexing, the unique molecular identifiers (UMIs) associated with each cellular index (cell) in R2C2 (top) and Illumina (center) datasets are shown as histograms. Cells are ranked by the number of UMIs and colored based on their rank in the R2C2 dataset. Red lines indicate cellular identifiers found in Illumina but not R2C2 data. At the bottom, the UMIs shared between cellular identifiers in Illumina and R2C2 datasets or unique to each dataset are shown as stacked histograms. Cells are ranked by the number of shared UMIs. Data for replicate 1 are shown

We extracted PBMCs from whole blood and processed the cells in replicate using the Chromium Single Cell 3′ Gene Expression Solution (10X Genomics) aiming to include 1500 cells each for two replicates. We then divided the full-length cDNA intermediate generated by the standard 10X Genomics protocol to perform both short- and long-read sequencing (Fig. 1A).

### Illumina data covers 10X-UMIs comprehensively

For sequencing on the Illumina NextSeq, we fragmented the full-length cDNA according to the standard 10X protocol. We demultiplexed and merged the resulting reads based on cellular barcodes and unique molecular identifiers (10X-UMIs) associated with every amplified transcript molecule during reverse transcription (see the “Methods” section). By only keeping transcript molecules with a raw read coverage of >3, we condensed 202,469,707 raw read pairs to 15,264,862 reads originating from the 3′ ends of unique transcript molecules across both replicates (~5000 molecules per cell).

### R2C2 data identifies the same cellular and molecular identifiers as Illumina data

For sequencing on the ONT MinION and PromethION sequencers, we processed 10ng of full-length cDNA using the previously published R2C2 workflow (see the “Methods” section). The resulting R2C2 libraries were then sequenced using standard ONT LSK-109 ligation-based sequencing kits. We processed the resulting ONT raw reads into R2C2 consensus reads using the C3POa pipeline (Table 1 and S1). We then merged reads in two sequential steps if they contained matching unique molecular identifiers (UMIs) in either the dsDNA splint used to circularize cDNA molecules (Splint-UMI) or the 10X oligo(dT) primer used to prime reverse transcription of poly(A) RNA molecules (10X-UMI).

**Table 1:** Read numbers throughout processing

	Basecalled reads	Post-processed R2C2 consensus reads	Splint-UMI merged R2C2 consensus reads	Splint/10X-UMI merged R2C2 consensus reads	Demultiplexed R2C2 reads
**Replicate 1**	29,529,179	11,564,494 (39.2%)	11,368,091 (98.3%)	7,853,440 (69.1%)	6,385,901 (81.3%)
**Replicate 2**	26,526,607	10,661,139 (40.2%)	10,276,420 (96.4%)	6,968,632 (67.8%)	5,652,620 (81.1%)

We generated 11,564,494 and 10,661,139 R2C2 consensus reads with average subread coverage of 3.04 and 1.89 for replicate 1 and replicate 2, respectively. We then merged 3.3% (Rep1) and 6.5% (Rep2) of this R2C2 consensus because their Splint-UMI identified them as originating from the circularization of the same cDNA molecule. Second, we merged 46.3% and 46.1% of these Splint-UMI merged R2C2 consensus reads in replicate 1 and replicate 2, respectively, because their 10X-UMI identified them as originating from the same RNA molecule. Across both replicates, this sequential merging process resulted in 14,822,072 Splint/10X-UMI merged R2C2 consensus reads (Table S2) with an average subread coverage of 3.73 (Additional file 1: Fig. S1), an average sequence length of 1358 bp (Additional file 1: Fig. S2), and median sequence accuracy of 98.0%.

Next, we demultiplexed these ~14.8 million Splint/10X-UMI merged R2C2 consensus reads based on the 10X cellular barcodes they contained. In this way, 81% of these reads could be successfully assigned to an individual cell, which compares favorably to the ~6% Illumina-independent and ~67% Illumina-guided assignment rates determined for standard ONT reads in previous studies [27, 31].

Moreover, 2974 (99.1%) of the 3000 cellular identifiers we determined independently from the R2C2 dataset also appeared in the Illumina dataset.

Because we merged reads in Illumina and R2C2 datasets based on the 10X-UMI, each read in either dataset should originate from a unique RNA molecule. Consequently, the number of unique molecules assigned to each cell was similar between the datasets, although the exhaustively sequenced Illumina dataset contained more molecules per cell than the non-exhaustive R2C2 dataset (Fig. 1B). Also, for each cell, 67% of the R2C2 reads contained a 10X-UMI that was also present in an Illumina read assigned to the same cell. Interestingly, the accuracy of R2C2 reads containing 10X-UMIs present in an Illumina read was significantly higher than the accuracy of R2C2 reads containing 10X-UMIs not present in an Illumina read (98.4% vs. 97.1%; *p* = 0.0 Monte-Carlo permutation test). This indicates that read accuracy plays an important role in accurately identifying UMI sequences. Although their RNA molecule of origin cannot be unambiguously identified, we chose to include these R2C2 reads in our downstream analysis, thereby valuing the extra information they might contain for isoform identification over their potential to distort the quantification of gene and isoform expression.

### Clustering single cells into cell types based on gene expression

We next investigated whether these R2C2 reads could be used to determine gene expression accurately enough to cluster single cells into cell types — an analysis step that is currently routinely performed using short-read-based gene expression. To this end, we used minimap2 to align R2C2 reads to the human genome (hg38) and used featureCounts to determine gene expression levels in each cell [32, 33]. For comparison, Illumina reads generated from the same cDNA were aligned using STAR and also processed using featureCounts [34]. Median Pearson *r* values for R2C2 and Illumina-based gene expression for the same cell showed a high correlation at 0.74 (Additional file 1: Fig. S3).

**Fig. 2:**
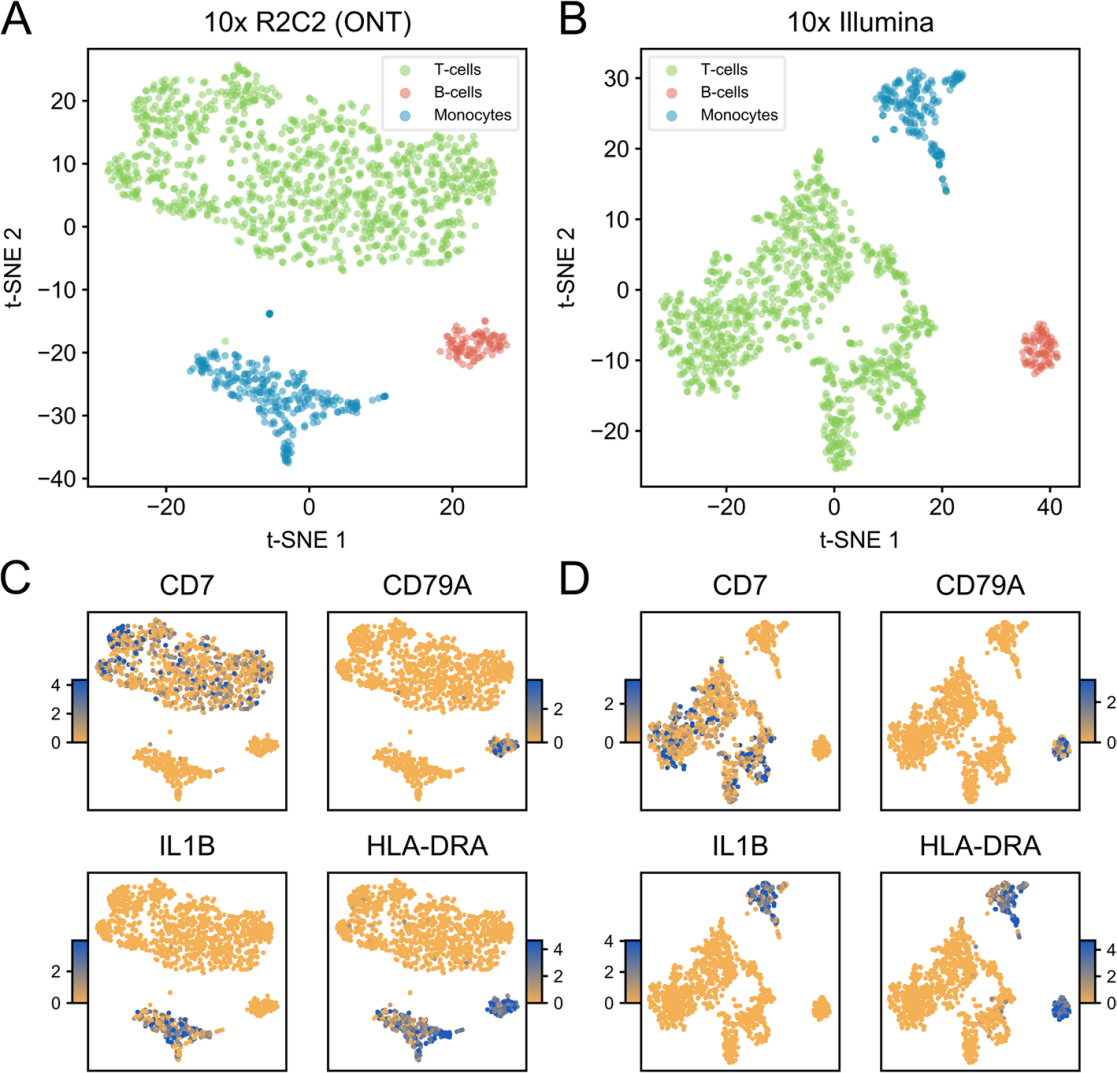
R2C2 and Illumina datasets independently cluster into B cells, T cells, and monocytes. Gene expression profiles were determined independently for each cell in R2C2 and Illumina datasets. The Seurat package was then used to cluster cells based on the gene expression profiles. The cells in R2C2 (**A**) and Illumina (**B**) datasets both clustered into 3 groups which, based on marker gene expression (**C** and **D**) could be identified as B cells, T cells, and monocytes. The color gradient (**C** and **D**) encodes ln(fold change), where the fold change is comparing that cluster’s expression to the rest of the data. Data for replicate 1 are shown

We then clustered R2C2 and Illumina datasets independently using the Seurat analysis package [35]. R2C2 and Illumina datasets both generated highly similar library metrics as determined by Seurat, i.e., genes (nFeatures) and molecules (nCount_RNA) per cell (Additional file 1: Fig. S4 and Additional file 1: S5). Seurat grouped cells in both datasets grouped into three cell type clusters. Based on marker gene expression, the major cell types could be identified as B cells (CD79A) [36], T cells (CD7) [37], and monocytes (IL1B) [38] — the expected composition of a PBMC sample (Fig. 2, Additional file 1: S6). Importantly, 99.4% of cells that were clustered in both datasets associated with the same cell type in the two datasets.

This showed that R2C2 reads show performance comparable to Illumina data for determining gene expression and clustering cell types in massively multiplexed single-cell experiments.

### Generating cell type-specific isoform-level transcriptomes

Having successfully sorted cells into cell types, we set out to generate high-quality transcriptomes for these cell types. This is possible because, as shown in previous studies analyzing 10X cDNA with long reads [27, 28], R2C2 reads appeared to cover entire transcripts (Additional file 1: Fig. S7).

First, as previously established [28], we pooled all reads associated with the cells of each cell type to create a synthetic bulk sample. We then identified transcript isoforms for each synthetic bulk cell type using Mandalorion [19–21, 29]. The majority (50–60%) of isoforms generated by Mandalorion for the individual cell types were classified by SQANTI [39] as either “full-splice-match” or “novel-in-catalog,” which represent likely full-length isoforms. This number increased to >80% if only multi-exon isoforms were considered. In aggregate, the cell type-specific isoforms we generated represent full-length B cell, monocyte, and T cell transcriptomes, with each transcriptome’s depths dependent on the number of cells and reads associated with each cell type (Table 2). With ~8.8 million R2C2 reads and 14,925 multi-exon isoforms, the T cell transcriptome is the most complete and likely most useful of the three cell types.

**Table 2:** Cell type-specific full-length transcriptome characteristics

Cell type	Number of cells	Number of reads	Number of genes with multi-exon isoforms	Number of multi-exon isoforms
B cells	179	625,334	1481 (plus 55 novel genes)	2006
T cells	2199	9,108,828	6934 (plus 448 novel genes)	14,925
Monocytes	464	2,042,162	2882 (plus 77 novel genes)	4530

### Differential isoform usage between cell types

In addition to determining which isoforms are expressed, we can also quantify the expression of these isoforms and investigate whether they are differentially expressed between the three cell types. To perform this differential isoform expression analysis, we first wanted to capture all the isoforms expressed in the entire dataset. To this end, we composed an additional “synthetic bulk” sample using the R2C2 reads from all cells in the dataset. We then used Mandalorion to identify all isoforms present in this “synthetic bulk” sample. In total, Mandalorion identified 17,010 isoforms at an average length of 1511 bp (Additional file 1: Fig. S1). Similar to the individual cell type isoform sets, the majority (66%) of isoforms in this synthetic bulk isoform set were classified by SQANTI to be either “full-splice-match” or “novel-in-catalog.” Importantly, the TSSs of 87% of all isoforms in this set had refTSS [40] support which gave us high confidence in their 5′ ends.

**Fig. 3:**
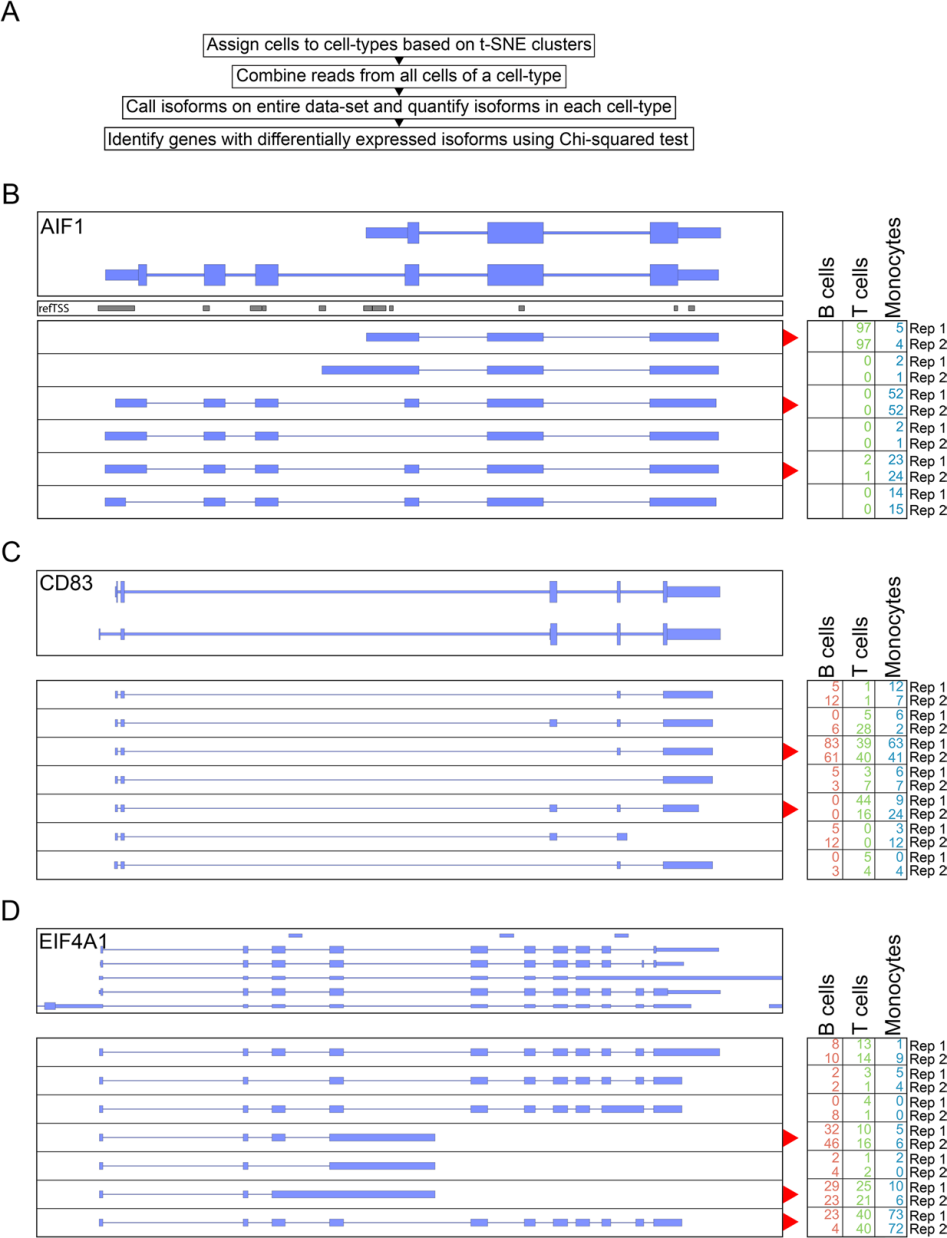
Identifying differentially expressed isoforms between cell types using clustered single-cell data. **A** Workflow of differentially expressed isoform identification. R2C2 reads are separated by cell type, then used to identify and quantify isoforms. Genes with differential isoform usage between cell types are then identified using chi-squared tests. **B**–**D** Genome Browser shots of three genes with differential isoform expression. Gene annotation is shown on top. Isoforms as determined by Mandalorion on the entire dataset are shown below (“top strand” = blue, “bottom strand” = yellow). Relative quantification (%) of each isoform in each cell type and replicate is shown on the right. Isoforms with the most variable changes in abundance are indicated with a red arrow. For AIF1, we also include a refTSS track

Next, we quantified the expression of each isoform in B cells, T cells, and macrophages. The quantified isoforms were then grouped by the genes they were associated with and genes with significant isoform usage between cell types were determined using a chi-square contingency table test. After filtering for genes expressed in at least two cell types and multiple testing correction, we identified 74 genes with differential isoform usage (*p*-value < 0.01) (Additional file 2: Table S3). The features that distinguished differentially expressed isoforms included alternative TSSs with refTSS support (AIF1, Fig. 3B), cassette exons (CD83, Fig. 3C), or poly(A) sites (EIF4A1, Fig. 3D).

### Isoform diversity is highly variably between genes

Next, we investigated whether single-cell-derived transcriptome information can enrich our understanding of isoform diversity. While pooling all reads associated with a cell type can serve as a basis for defining transcriptome annotations, this approach loses information on which isoforms are expressed by which individual cell and due to coverage cut-offs likely presents a conservative estimate of the true isoform diversity present in a cell type.

In the 3000 cell dataset we present here, we have sufficient coverage to generate isoforms for each cell independently. Using Mandalorion, we generated a median of 127 multi-exon isoforms per cell, with the majority being classified as either “full-splice-match” (77%) or “novel-in-catalog” (11%).

We then analyzed isoform diversity across ~3000 cells in the dataset. To this end, we merged identical isoforms expressed by different cells. We then determined how many cells expressed isoforms for any given gene.

**Fig. 4:**
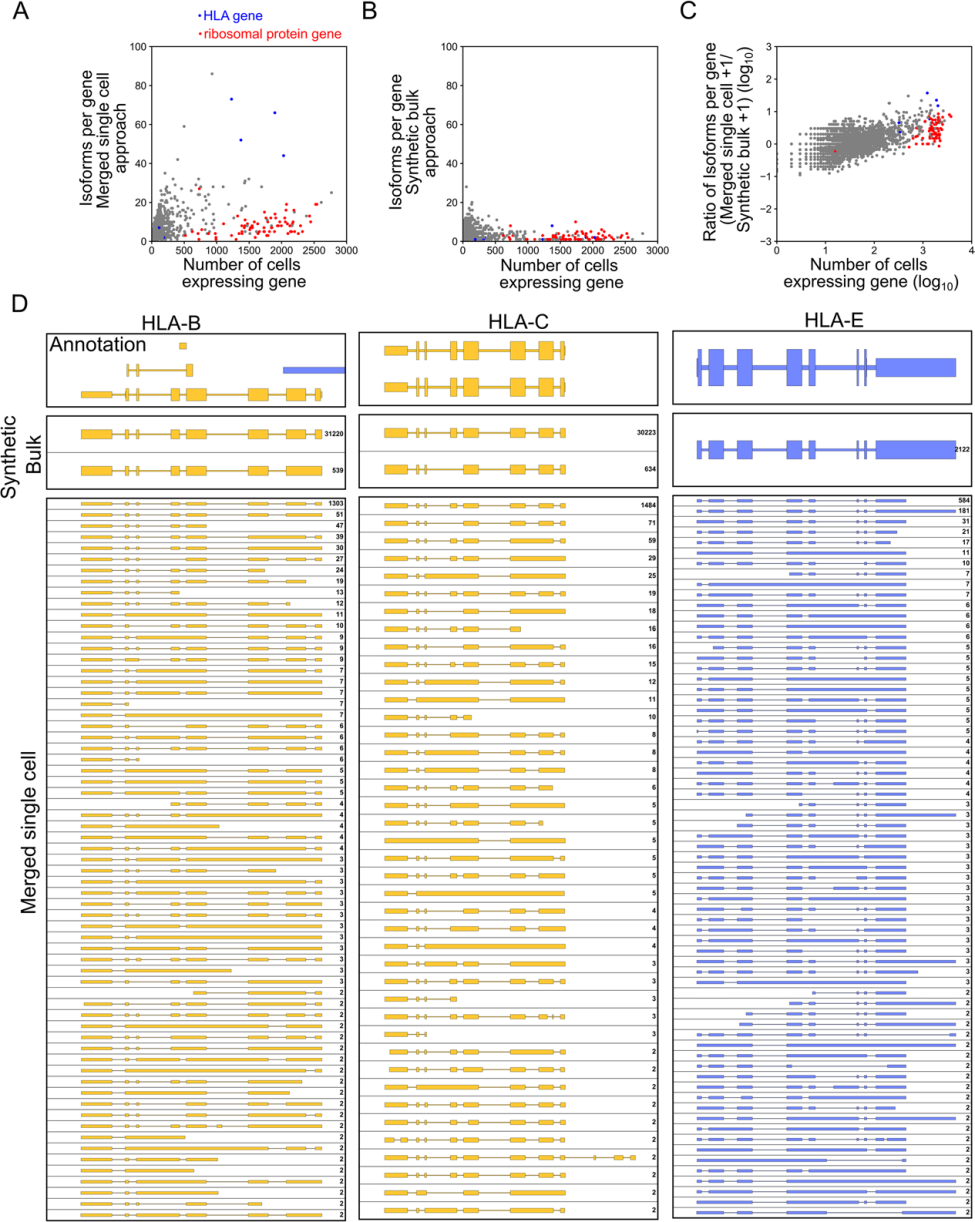
Genes show a wide range of isoform diversity. We generated an isoform-level transcriptome for each cell in our dataset and then analyzed the isoform diversity for different genes by merging these isoforms. **A** The correlation of the number of cells expressing an isoform for a gene and how many unique isoforms we identified for that gene using the “merged single cell” approach is shown as a scatter plot. **B** The correlation of the number of cells expressing an isoform for a gene and how many unique isoforms we identified for that gene using the synthetic bulk approach is shown as a scatterplot. **C** The correlation of the number of cells expressing an isoform for a gene and the ratio of the number of isoforms identified for that gene with the “merged single cell” and “synthetic bulk” approaches. Both number of cells and isoform ratio are shown as log_10_. **A**–**C** Genes encoding ribosomal proteins and HLA proteins are shown in red and blue, respectively. **D** Genome Browser shots HLA genes are shown. Genome annotation is shown on top, isoforms determined by the synthetic bulk approach in the middle, and isoforms determined by the merged single-cell approach at the bottom (“top strand” = blue, “bottom strand” = yellow). A number of reads (synthetic bulk) or cells (merged single cells) associated with an isoform are shown on the right

Interestingly, much of the single-cell isoform diversity we observe seemed to be based on intron retention and/or be incompletely spliced transcripts and varied greatly between genes (Fig. 4A). On one end of the spectrum, genes encoding ribosomal proteins in particular are expressed in the majority of cells, yet we identify few unique isoforms for these genes. For example, 1299 cells expressed a total of 1299 isoforms (as determined by Mandalorion) of the ribosomal protein gene RPL35. After merging all identical isoforms, only 8 unique isoforms remained and only one of those was expressed by more than one cell. On the other end of the spectrum, genes like LMNA are also expressed by a majority of cells but feature many unique isoforms. In fact, 930 cells expressed a total of 969 unique LMNA isoforms. After merging all identical isoforms, only 305 unique isoforms remained and 86 of those were expressed by more than one cell.

Unique isoforms expressed by more than one cell as determined by this “merged single cell” approach could therefore be used to enrich isoform annotations based on bulk or synthetic bulk data. For example, combining all R2C2 reads collected for all the cells in this study and identifying isoforms based on this synthetic bulk yielded one isoform for RPL35 but also only 3 isoforms for LMNA, likely due to minimum relative abundance requirements of 1% at a locus set as default in Mandalorion. In fact, most genes expressed by many cells had a low number of isoforms identified by the “synthetic bulk” approach (Fig. 4B).

By systematically comparing the “merged single cell” and “synthetic bulk” approaches, we showed that the number of cells expressing an isoform in the “merged single cell” approach and the number of reads associated with that isoform in the “synthetic bulk” approach correlated well (Pearson’s *r* = 0.71, Additional file 1: Fig. S8). We also found that the more cells expressed isoforms for a gene, the more likely the “merged single cell” approach was to identify additional isoforms. This analysis highlighted the behavior of HLA class I genes, in particular HLA-B, HLA-C, and HLA-E (Fig. 4C), which all showed >40 isoforms with the “merged single cell” approach but only one or two in the “synthetic bulk” approach (Fig. 4A, B, D).

### Extracting paired adaptive immune receptor sequences from B and T cells

In addition to the analysis of regular transcript isoforms, we investigated whether our datasets enable the identification and pairing of adaptive immune receptor (AIR) transcripts. AIR transcripts encode for antibodies and T cell receptors which pose unique challenges for sequencing applications. Each antibody (IG) or T cell receptor (TR) is encoded by two AIR transcripts each of which is transcribed from a gene whose V (, D,) and J segments are uniquely rearranged in each individual B or T cell.

**Fig. 5:**
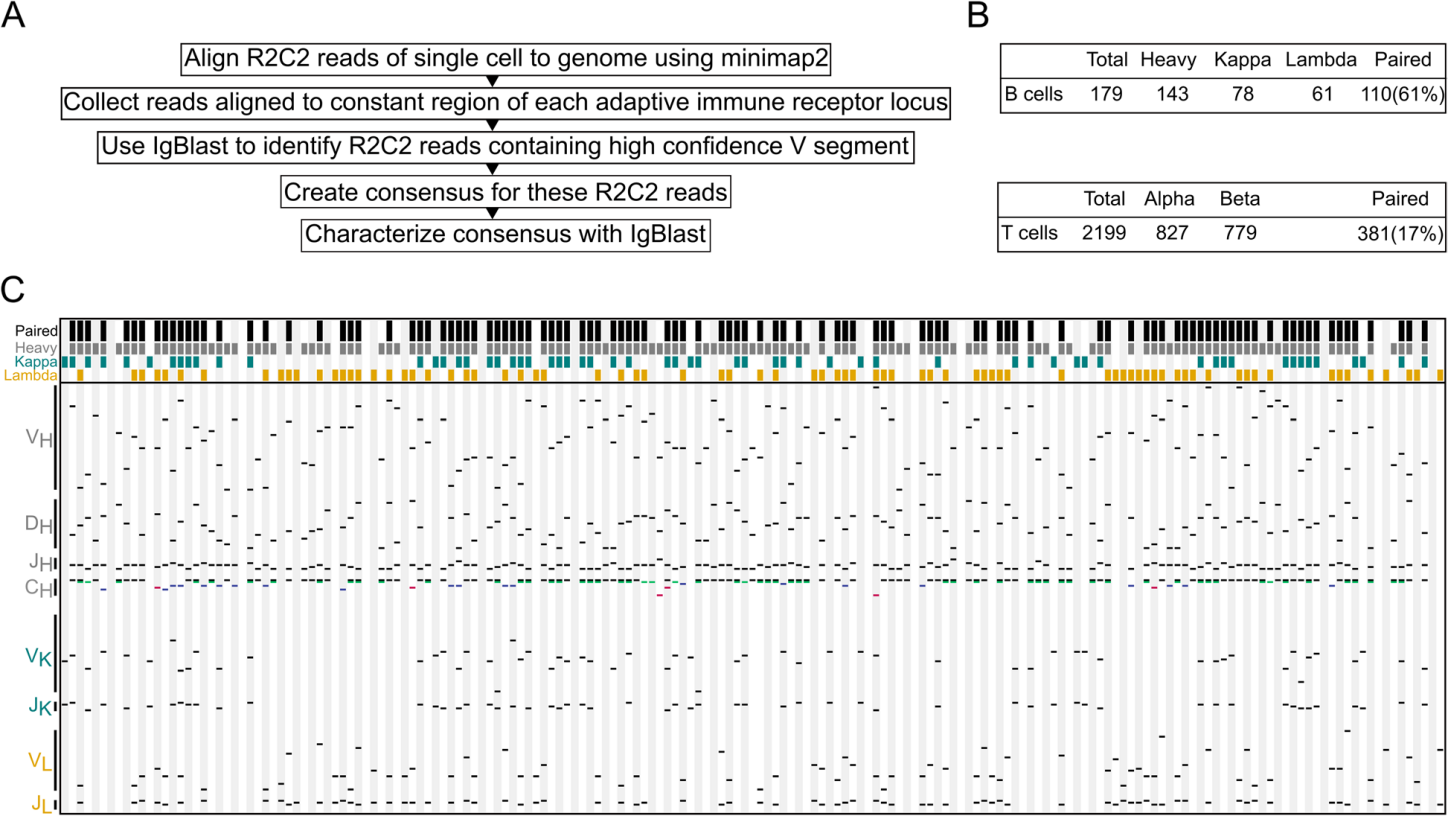
IG and TCR transcripts can be identified and paired in 10X R2C2 data. **A** The workflow to identify antibody (IG) and T cell receptor (TCR) transcripts for each individual cell. **B** Numbers of cells for which IG or TCR transcripts could be identified and paired. **C** Schematic of IG identification, composition, and pairing. Each column represents a single B cell. Colored blocks on top of each column indicate whether a cell contains paired IG transcripts (black) and whether an IGH (heavy: gray), IGK (kappa: teal), or IGL (lambda: orange) transcripts were detected. Below the diversity of the detected sequences is shown. Black lines indicate which gene segments were used when an IG sequence was recombined from the germline genome. In C_H_, it is also shown which isotype(s) we detected (IGHM: black, IGHD: green, IGHA1 or 2: red, IGHG1-4: blue) for each cell

Our standard Mandalorion transcript isoform identification workflow does not capture these AIR transcripts reliably because it relies on read alignments which fail for the highly repetitive and rearranged IG heavy (IGH), IG light (IG kappa (IGK) and lambda (IGL)), TCR alpha (TRA), and beta (TRB) loci. To capture AIR transcripts reliably, we first identified R2C2 reads which aligned to the constant region exons in the IG and TR loci. We then determined which of these reads contained a high-quality V segment using IgBlast [41]. Finally, we used these filtered reads to determine consensus sequences for each locus and cell (Fig. 5A).

For many B cells, we determined multiple sequences for different isotypes (IGHM, IGHD, IGHG (1, 2, 3, and 4), and IGHA (1 and 2)) (Additional file 2: Table S4) and isoforms (membrane bound and secreted). In the vast majority of cases (103/108) (Fig. 5B), transcripts contained the same V segment, indicating that they represent alternative splicing products of the same rearrangement. We succeeded in determining paired IG sequences for 110 B cells and 381 T cells which represent 61% and 17% of all B and T cells analyzed in this study, respectively (Fig. 5C). Importantly, as would be expected for a random sample of B cells, the V (, D,) and J segment usage composition of the paired transcripts of these cells was highly diverse (Fig. 5C).

## Discussion

Here, we present modified molecular biology workflows and new computational tools that make it possible to apply the R2C2 method to full-length single-cell cDNA pools generated by the droplet-based 10x Genomics Chromium controller. We processed 10ng of cDNA generated as an intermediate product of the 10X Genomics Chromium Single Cell 3′ Gene Expression Solution into R2C2 sequencing libraries. We sequenced these libraries and demultiplexed the resulting data to produce over 12 million unique transcript molecules generated from ~3000 PBMCs. This amounted to ~4000 R2C2 reads per cell as opposed to the 20,000 Illumina reads 10x Genomics recommends. At this coverage, low expressed genes are likely to be excluded from differential gene and isoform analysis. We nonetheless used these single-cell data to determine monocyte, T cell, and B cell clusters; generate isoform-level transcriptomes for these cell types; investigate single-cell isoform diversity; and pair adaptive immune receptor transcripts.

The ability to analyze the full-length transcriptomes of single cells without the need for Illumina short-read data has the potential to simplify experimental workflows. The ability to perform this analysis on low-cost ONT sequencers will make it more accessible. This is made possible through the use of the R2C2 sample preparation method which can increase the base accuracy of ONT MinION sequencers to ~99%. In this study, the R2C2 base accuracy was closer to 98% due to shorter raw reads. We aimed for shorter raw reads to increase R2C2 read numbers and, to this end, reduced the stringency of our size selection prior to sequencing (Table S1).

Outside of R2C2, raw nanopore reads are becoming more accurate and are used to analyze 10X cDNA with the help of Illumina data or by themselves using modified 10X protocols with longer cell barcodes and UMI sequences. Furthermore, single-cell studies using the PacBio Sequel II, while limited in overall throughput and hampered by per-read cost of the sequencer, benefit from the very high accuracy of the reads which simplifies computational analysis. Going forward, the trade-off between throughput, cost, and accuracy of ONT MinION and PromethION as well as PacBio Sequel II sequencers will have to be considered closely and the best compromise may well vary between studies.

At current throughput and accuracy, the combination of ONT sequencers and the R2C2 method allows the analysis of thousands of cells. An increase in read output will make it possible to either analyze more cells or sequence all transcripts reverse transcribed by the 10X Genomics workflow. In the current study, with about 4000 R2C2 reads per cell, we captured about 67% of the molecules present in an exhaustively sequenced Illumina dataset of the same cDNA. This was sufficient to cluster cell types and generate single-cell transcriptomes. As of now, potential users of this technology will have to decide whether to include these unmatched molecules in their downstream analysis. While these molecules are likely to help define isoforms by increasing read depth, they might also distort gene and isoform quantification. An increase in accuracy would make future demultiplexing and UMI merging steps more efficient and hopefully increase UMI matching rate to a point where this decision does not have to be made and different treatments and conditions can be safely compared across experiments.

The demultiplexing method we developed generates a pre-filtered list of the most common barcodes in a cDNA pool and then compares each R2C2 read’s cellular barcode to this list. This is a more efficient and straightforward approach than comparing UMI sequences across all R2C2 reads. Furthermore, our demultiplexing strategy can handle sequencing errors (see the “Methods” section), yet, at 98% read accuracy, it still only manages to demultiplex ~81% of R2C2 reads. This is better than previously published approaches, but not ideal [27, 31]. Increasing accuracy to the level of PacBio Iso-Seq [23, 24, 42] could increase this number significantly. Paired with the higher throughput we can achieve by optimizing raw read to consensus read conversion as we have previously shown [43], future experiments could only retain UMIs which were observed more than once, similar to how we analyze Illumina data (see the “Methods” section).

Beyond improving the R2C2 method itself, a tempting approach would of course be to use Illumina short-reads to aid the cell barcode and UMI sequence assignment [27]. Furthermore, any error/indel-prone long-read method could benefit from a redesign of cell barcode and UMI sequences present in the oligos used by the 10X Genomics workflow. A recent example of a long-read appropriate design [25] used homodimeric nucleoside phosphoramidite building blocks to synthesize cellular barcodes and UMI sequences composed of sequence dimers to improve demultiplexing and molecule assignments.

The question remains whether cell barcodes and UMI sequences can be improved for long-read sequencers with more subtle changes not requiring specialized oligo synthesis. Currently designed exclusively for short-read sequencers, both 16nt cell barcode and 10nt UMI sequence are present in the 10X oligodT primer directly adjacent to each other. Cell barcode and UMI sequence can therefore only be parsed from a sequencing read based on their sequence distance from the constant part (PCR priming site) of the oligodT primer. This means that an indel in the cell barcode will also affect the UMI sequence next to it, thereby aggravating the consequences of the most common long-read sequencing error type. This is made even more problematic because the UMI sequence is directly adjacent to the actual oligodT stretch of the oligodT primer — a long stretch of Ts which is notoriously hard for long-read sequencers to get right and will also likely affect the sequences adjacent to it.

We propose that future iterations of the oligodT primer contain spacer sequences of known length and sequence at defined positions between and within the cell barcodes and UMI sequences.

Instead of and oligodT primer with the following structure:

[PCR_priming_site]XXXXXXXXXXXXXXXXNNNNNNNNNNTTTTTTTTT

with X denoting variable bases of the cell barcode, N variable bases of the UMI sequence, and T actual T bases of the oligodT primer, we propose a oligodT primer structure as follows:

[PCR_priming_site]XXXXX**ATA**XXXXX**TAT**XXXXXX**CTC**NNNNN**GAG**NNNNN**ACA**TTTTTTTTT

where the bolded A, T, C, and G bases create sequence spacers that can be used to easily parse cell barcodes and UMI sequences as well as immediately detect and mitigate indel errors. Because read positions with the exact same base in all sequenced molecules can be problematic for Illumina sequencers, there could be 4 different combinations of these spacers to make sure the read positions they occupy have a balanced base composition.

In its current state, the 10X/R2C2 method we developed allowed us to generate isoform-level transcriptomes for monocyte, B cell, and T cell populations. Because the different cell types were present in the analyzed PBMC sample at varying frequencies, monocyte, B cell, and T cell transcriptomes contained reads derived from varying numbers of cells. For the B cell transcriptome, we used 625,334 reads derived from 179 B cells. Rarefaction analysis of full-length transcriptome sequencing in previous bulk experiments [44] strongly suggests that this B cell transcriptome is almost certainly not saturated and sequencing more cells would result in a much more exhaustive isoform-level transcriptome. On the other hand, for the T cell transcriptome, we used 9,108,828 reads derived from 2199 T cells. However, rarefaction analysis of full-length transcriptome sequencing of bulk RNA from a lymphoblastoid cell line [45] again suggests that even this sequencing depth might not yield a transcriptome at saturation.

Because the number of unique molecules generated per single cell is limited (~around 5000 in this study), increasing sequencing depth to reach transcriptome saturation in future single-cell studies will have to be accomplished by increasing the number of cells sequenced. The relative frequencies of specific cell types therefore will have to be taken into account when determining how many total cells to include in a single-cell experiment if the goal is to generate comprehensive transcriptomes for these specific cell types. The exact number of reads required to reach saturation will depend on cell type/state, the sequencing method, and the isoform-calling pipeline, but based on bulk studies will likely be above 10 million, which corresponds to more than 2000 cells.

We then used a framework developed for a previous study [30] to show that these cell types show differential isoform expression. The ability to identify differentially expressed isoforms expands the quality of information that can be extracted from single-cell experiments and opens the door to a much more nuanced understanding of gene regulation.

Beyond investigating isoform expression on the cell type level, we investigated the extent of isoform diversity on the single-cell level. While some genes showed low isoform diversity, i.e., most cells express the same isoform, some genes showed high diversity, i.e., many cells express unique isoforms. This wide range of isoform diversity will pose a formidable challenge for single-cell-level differential isoform expression analysis going forward. Future studies into how this wide range of isoform diversity is maintained and used by cells are bound to generate fascinating insights into transcript processing and cellular function.

In the meantime, using isoforms identified independently for single cells can already inform isoform identification. While different isoform identification tools like TALON [46], FLAIR [47], or StringTie2 [48], and Mandalorion use different strategies when identifying and filtering isoforms, they all rely on some form of read coverage cut-off to differentiate real isoforms from the noise produced by any sequencing method. However, PCR or sequencing artifacts generated within a single cell can overcome these cut-offs and result in the false-positive identification of isoforms. The information of how many single cells express an isoform could therefore aid in the identification of real or biologically meaningful isoforms as each single cell can be seen as an independent biological replicate.

Finally, taking advantage of the single-cell nature of this dataset, we performed analysis on the most complex part of T cell and B cell transcriptomes, namely adaptive immune receptor transcripts. By sequencing and pairing adaptive immune receptor transcripts expressed by single T and B cells, we showcased the power of long reads for resolving even the most challenging transcript isoforms — without the need for specialized protocols [31]. This will be of particular use when analyzing complex samples that contain, but are not limited to, immune cells like solid or liquid tumors.

## Methods

### Single-cell cDNA library preparation

Full-length cDNA pools and Illumina libraries were prepared by 10X Genomics. PBMCs were sourced from Stemcell Technologies and prepared for sequencing using the 10X Genomics Chromium Single Cell 3′ Gene Expression Solution. Preparation of the cDNA was done according to the manufacturer’s instructions with the exception of the extension time for the final PCR reaction which was standard 1 min for replicate 1 but increased to 4 min for replicate 2.

### Illumina sequencing and read processing

Illumina libraries were sequenced on the Illumina NextSeq with Read1 = 26bp and Read2 = 134bp.

Overall, a NextSeq flowcell generated 107,911,006 reads for replicate 1 and 75,753,410 reads for replicate 2. Reads were then demultiplexed and collapsed by determining the 1500 most frequent cellular barcodes, perfectly matching cell barcodes to the most frequent, and then filtering for unique cell barcode/10X-UMI combinations.

Reads for each cell were then aligned to the human genome (hg38) using STAR (*--runThreadN 30 --genomeDir /path/to/STAR/index/ --outSAMtype BAM SortedByCoordinate --readFilesIn /path/to/reads --outFileNamePrefix /path/to/alignment/dir*).

### Nanopore sequencing and read processing

Full-length cDNA pools were prepared as described previously. In short, 10ng of cDNA is circularized using a DNA splint compatible with 10X cDNA and the NEBuilder HIFI DNA Assembly Master Mix (NEB). The DNA splint was generated by primer extension of the following oligos:

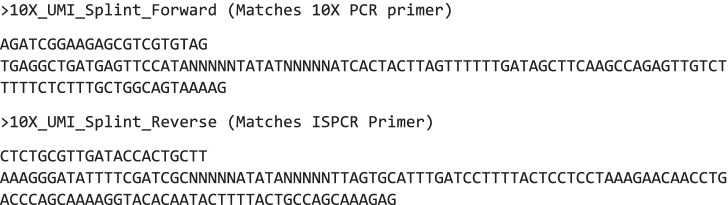



Non-circularized DNA is digested using exonucleases I and III and lambda. Circularized DNA is amplified using rolling circle amplification using Phi29 (NEB). The resulting HMW DNA is debranched using T7 Endonuclease (NEB) and purified and size-selected using SPRI beads. This DNA containing concatemers of the originally circularized cDNA is then sequenced using the LSK-109 kit on either ONT MinION or PromethION sequencers (Table S1). The resulting raw reads were processed into consensus reads using the C3POa pipeline (v2.2.2). All consensus reads were then assigned a cell of origin. In a first step, we determined the most common ~1500 cellular identifiers in our sample using a simple counting strategy. Then, we assigned reads to the most similar cellular identifiers if they fit the following criteria:

*1.) L1 < 3*

*and*

*2.) L1 < L2 - 1*

*where L1 is the Levenshtein distance between the read’s cellular identifier and the most similar known cellular identifier and L2 is the Levenshtein distance between the read’s cellular identifier and the second most similar known cellular identifier.*

These consensus reads were demultiplexed based on their cell assignment, and they were merged if they contained the similar UMIs in their splint back-bones using the ExtractUMIs and MergeUMIs utilities (https://github.com/rvolden/10xR2C2). The resulting reads were then merged again if they contained the similar 10X-UMIs in their adapters using the ExtractUMIs and MergeUMIs utilities (https://github.com/rvolden/10xR2C2).

The resulting Splint/10X-UMI merged R2C2 consensus reads were then demultiplexed based on their initial cell assignments. If a Splint/10X-UMI merged, R2C2 consensus read was generated by merging reads with different cell assignments it was discarded. Reads for each cell were then aligned to the human genome (hg38) using minimap2 [32] (*-ax splice --secondary=no -G 400k*).

### Cell type clustering

Both Illumina and R2C2 data were analyzed in the same way independently. First gene expression tables were generated using featureCounts [33]. Then, these tables were parsed for input into the Seurat R package (v3) [35]. Seurat generated cell type clusters using the following main settings (*min.cells=3, min.features=200, percent.mt<5, 2500>nFeature_RNA>200, nfeatures=2000, dims=1:10, resolution=0.08 (0.08 used for nanopore, 0.03 for Illumina), log normalization, and vst selection*).

For each cell, cell type information was extracted based on location for downstream analysis.

### Isoform analysis

We generated high confidence isoforms using the latest version of the Mandalorion pipeline (Episode III.5, https://github.com/rvolden/Mandalorion).

#### Cell type transcriptomes

All reads and subreads assigned to cells of a cell type were pooled. Mandalorion was run on these files with the following settings:

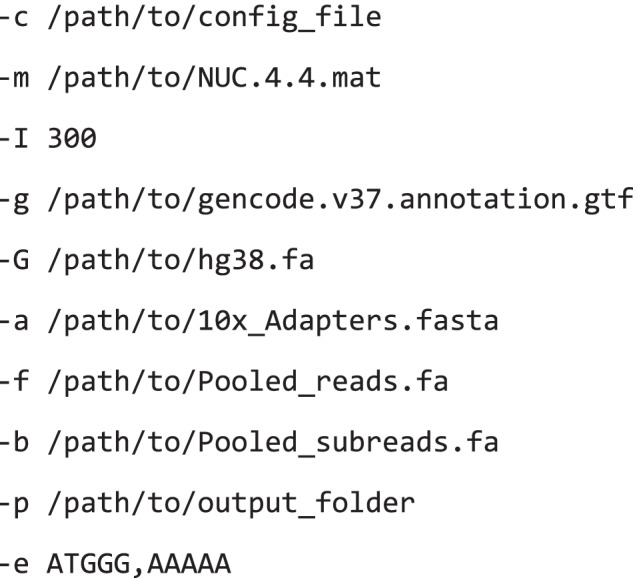



with 10x_Adapters.fasta containing the following sequences:

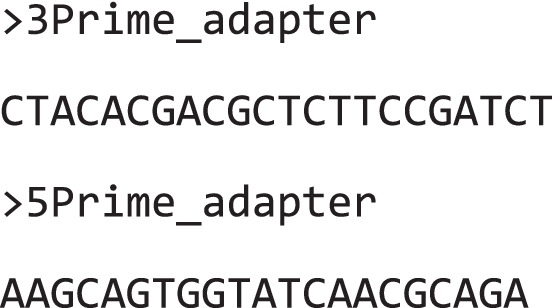



#### Single-cell transcriptomes

Mandalorion was run on the reads, read alignments, and subreads of each individual cell. Mandalorion was run with the following settings:

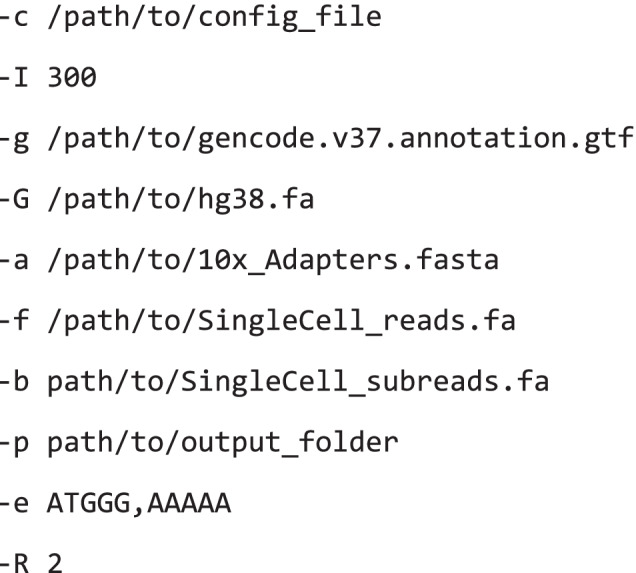



Note that we reduced the minimum number of reads required to identify an isoform to 2.

The resulting isoform psl files were converted to gtf files and classified using the sqanti_qc.py program and the following settings:

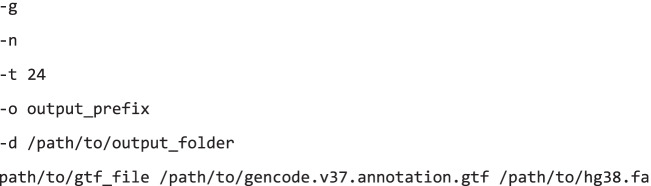



#### Isoform diversity analysis

Similar isoforms were merged using the merge_psls.py utility which accepts a list of isoform fasta and psl files and merges isoforms if they:

1. Use all the same splice sites
  This step is base-accurate but will treat splice site a single base pair apart as equivalent if one site is much less abundant than the other
2. Use the similar start and end sites
  This step will consider sites similar if they are at most 10nt apart. Because isoforms are iteratively grouped at this step, individual isoforms in a merged group might have sites that are further than 10nt apart but are connected by a third isoform between them.

### Adaptive immune receptor analysis

For each cell, reads aligning to the T cell or B cell receptor loci were extracted from sam files using samtools view [49] and the below genomic coordinates.

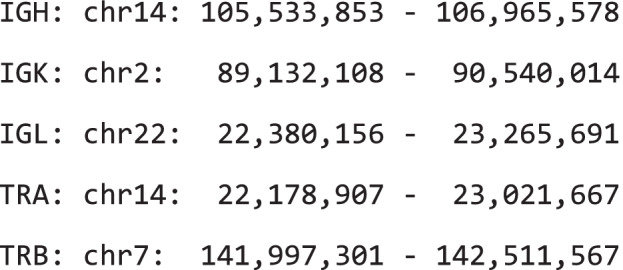



Reads were then analyzed for each cell and locus (and for IGH, each isotype/isoform) separately by filtering reads for a high-quality match to a V segment retrieved from IMGT [50] using IgBlast [41] and the following settings:

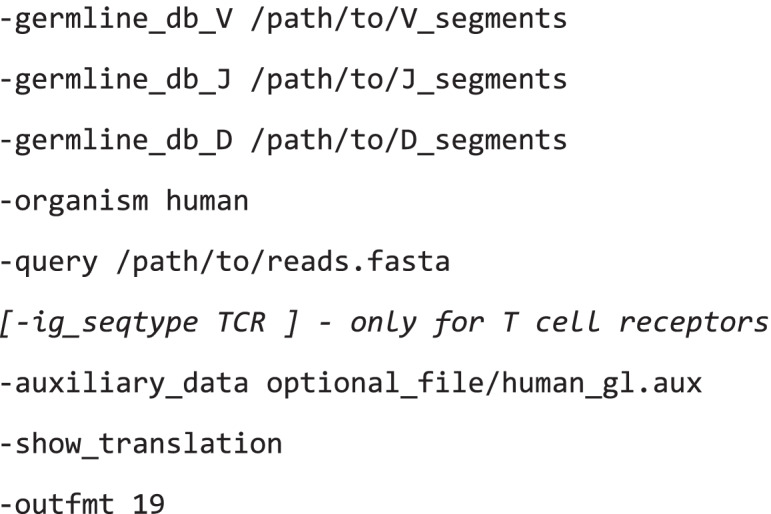



Filtered reads for each cell were then used to generate consensus reads for each locus. Those consensus reads were then assigned V, (D,) and J segments using IgBlast and the same settings as above. All scripts used for this analysis and a wrapper script automating this analysis are available at https://github.com/christopher-vollmers/AIRR-single-cell.

## Supplementary Information

**Additional file 1.** Supplemental figures 1-8 and tables 1-2.

**Additional file 2.** Supplemental tables 3-4. (XLS 169 kb)

**Additional file 3.** Cell type specific isoforms in psl format.

**Additional file 4.** Peer review history.

## Data Availability

We uploaded all data generated for this study to the SRA where it is available under BioProject accession PRJNA599962 [51]. B cell, T cell, and monocyte transcriptomes are available in the Additional file 3 archive. We have made the code required to demultiplex R2C2 reads and format gene expression matrices for Seurat available on GitHub: https://github.com/rvolden/10xR2C2 [52], 10.5281/zenodo.5826346 [53] Code for AIRR analysis is also available on GitHub: https://github.com/christopher-vollmers/AIRR-single-cell [54], 10.5281/zenodo.5814074 [55]
