# Supplementary material for: Single-cell isoform analysis in human immune cells
Source: Genome Biol. 2022 Feb 7;23:47. doi: 10.1186/s13059-022-02615-z (PMC8819920; doi:10.1186/s13059-022-02615-z)
Supplement: Supplementary file 1 — Additional file 1. Supplemental figures 1-8 and tables 1-2. [file 13059_2022_2615_MOESM1_ESM.pdf]

Supplementary Material to

Single-cell isoform analysis in human immune cells

by

Roger Volden and Christopher Vollmers

Table of Contents:

Supplementary Figures S1-S8
Supplementary Table S1-S2

Supplementary Tables S3 and S4 in separate .xls file

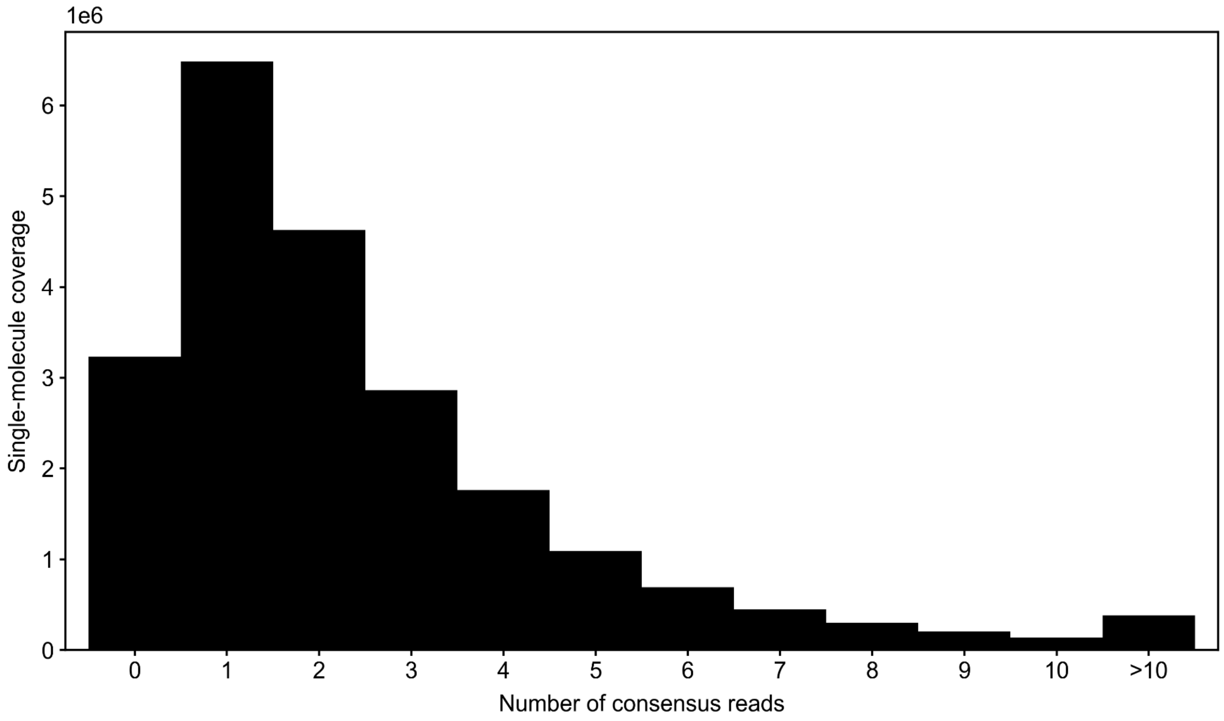

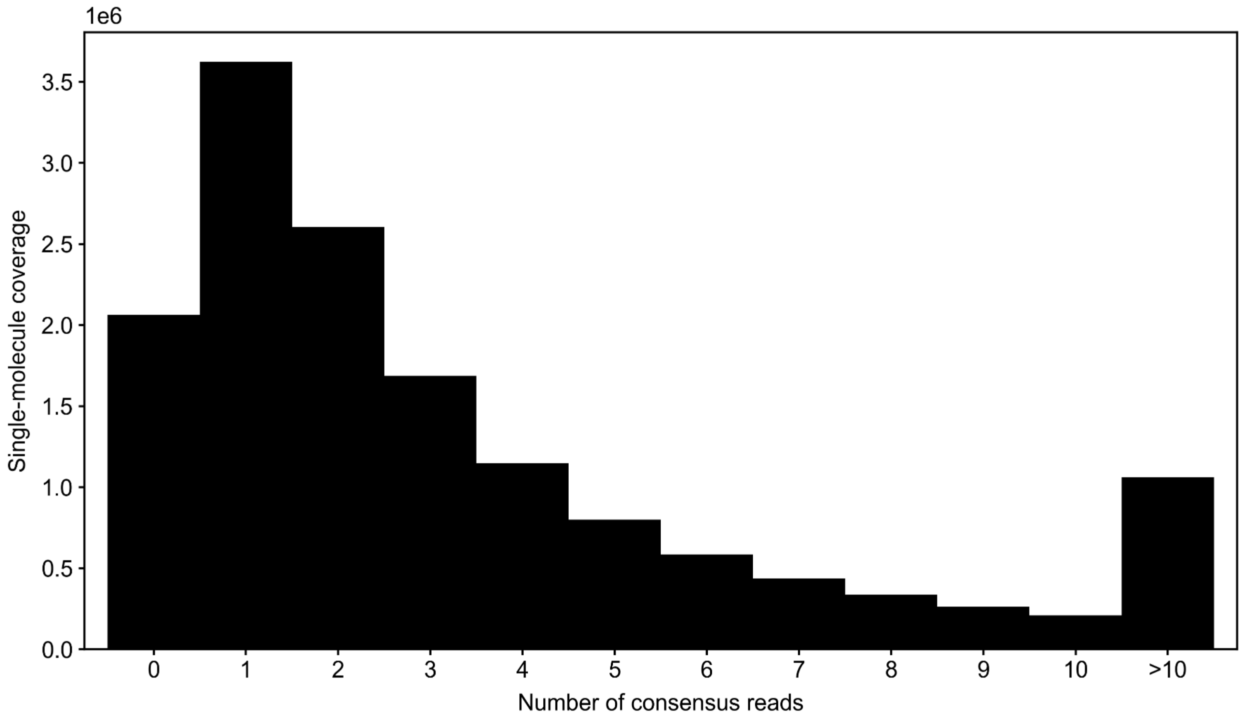

Supplementary Figure S1: Subread coverage per molecule. Top: subread coverage per molecule before merging splint and 10x UMIs. Bottom: subread coverage per molecule after merging splint and 10x UMIs. Molecules with 0 subreads are assembled from overlapping incomplete subreads.

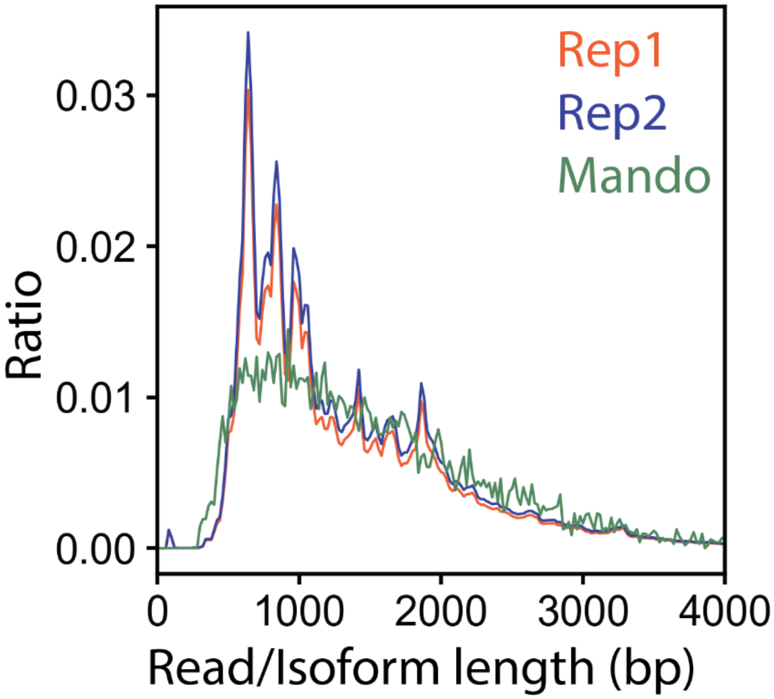

Supplementary Figure S2: R2C2 read and Mandalorion isoform length distribution. Read and isoform length distributions (as determined by Mandalorion for the “synthetic bulk” dataset) were determined by the length of the fasta sequence entries in the respective files.

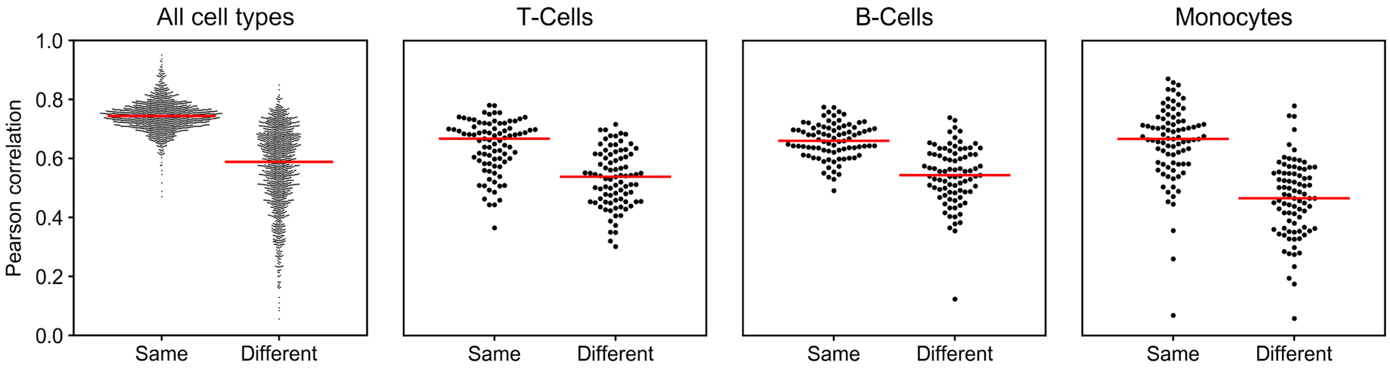

Supplementary Figure S3: Swarm plots of gene expression correlation between R2C2 and Illumina. The median Pearson correlation for each swarm is shown in red. From left to right: (All cell types, same) cells were matched based on their cellular barcode from R2C2 and Illumina. (All cell types, different) R2C2 cells were correlated to a random cell in the Illumina data. The next three swarms were subsampled to 85 points because there are 89 B-Cells. (T-Cells, same) Random T-Cells were correlated between R2C2 and Illumina data. (T-Cells, different) Random R2C2 T-Cells were correlated with random Illumina non-T-Cells. (B-Cells, same) Random B-Cells were correlated between R2C2 and Illumina. (B-Cells, different) Random R2C2 B-Cells were correlated with random Illumina non-B-Cells. (Monocytes, same) Random Monocytes were correlated between R2C2 and Illumina. (Monocytes, different) Random R2C2 Monocytes were correlated with random Illumina non-Monocytes.

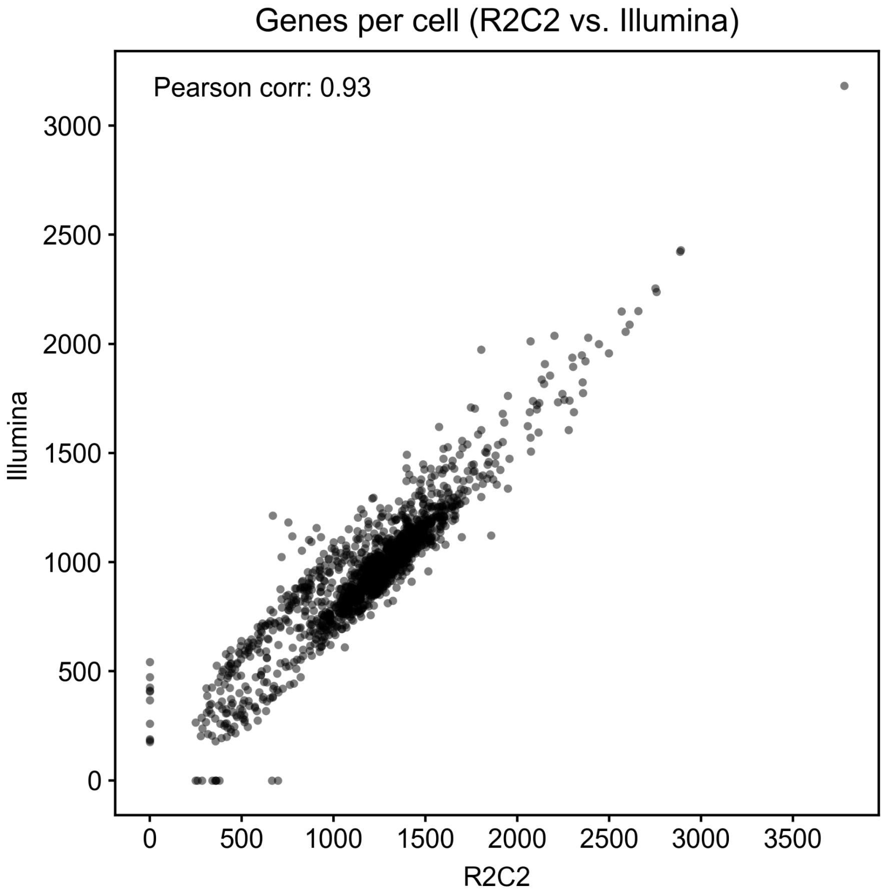

Supplementary Figure S4: Genes per cell in R2C2 and Illumina. The number of genes expressed by each cell as determined by R2C2 and Illumina is shown as a scatter plot.

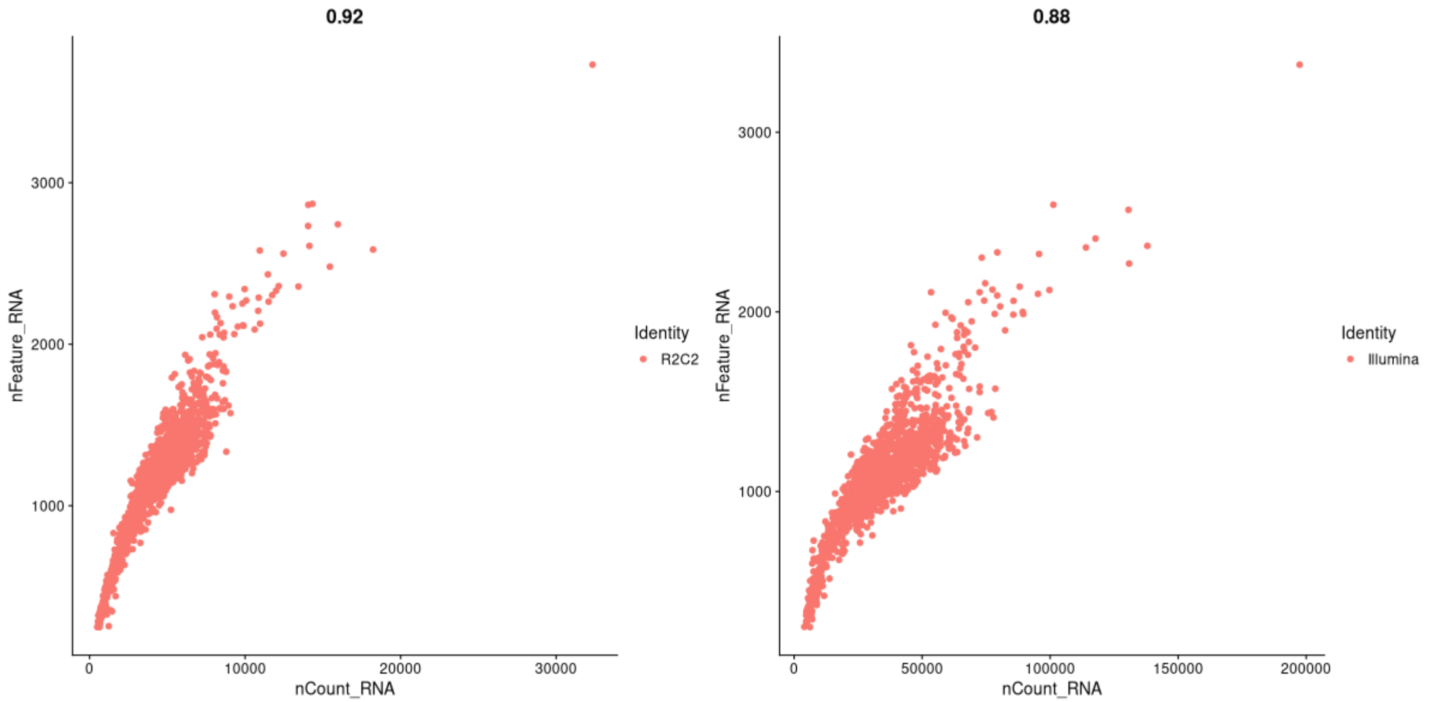

Supplementary Figure S5: Number of molecules vs. number of genes per cell. The number of molecules (nCount_RNA) was plotted against the number of genes (nFeature_RNA) for each cell as scatter plots for both RNA and Illumina datasets. Pearson's r values are shown on top of each plot.

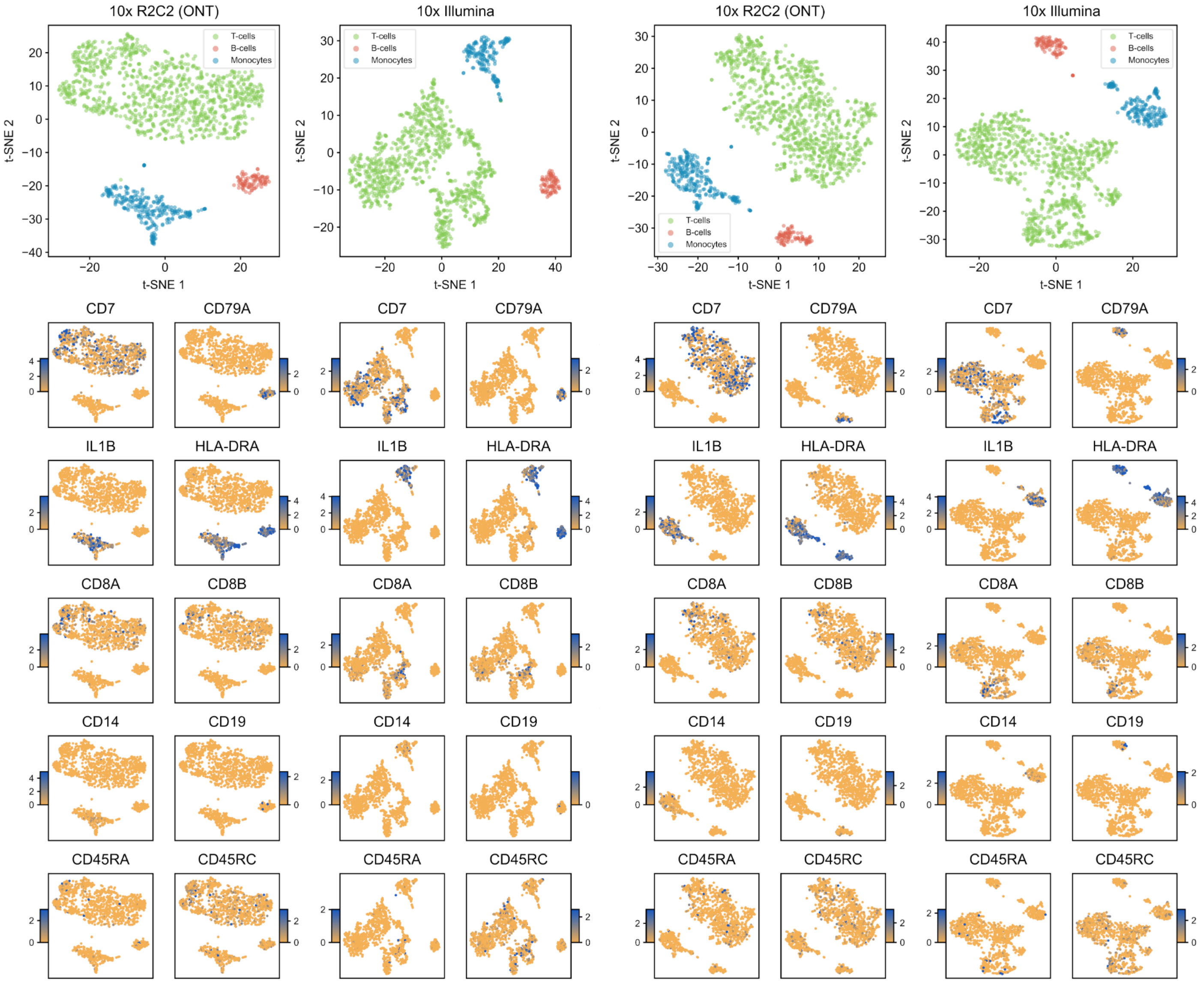

Supplementary Figure S6: t-SNE plots with additional marker genes for replicates 1 and 2. As for Figure 2 plots are based on gene expression data as calculated by featureCounts and Seurat. Plots for replicate 1 and replicate 2 are shown on the left and right respectively. Top left: replicate 1 cell type clusters for R2C2 and Illumina. Bottom left: replicate 1 expression heat maps for various marker genes where the two columns on the left are for R2C2 and the right two are Illumina. Top right: replicate 2 cell type clusters for R2C2 and Illumina. Bottom right: replicate 2 expression heat maps for various marker genes where the two columns on the left are for R2C2 and the right two are Illumina. Additional marker genes taken from [14]. The color gradient encodes ln(fold change), where the fold change is comparing that cluster's expression to the rest of the data.

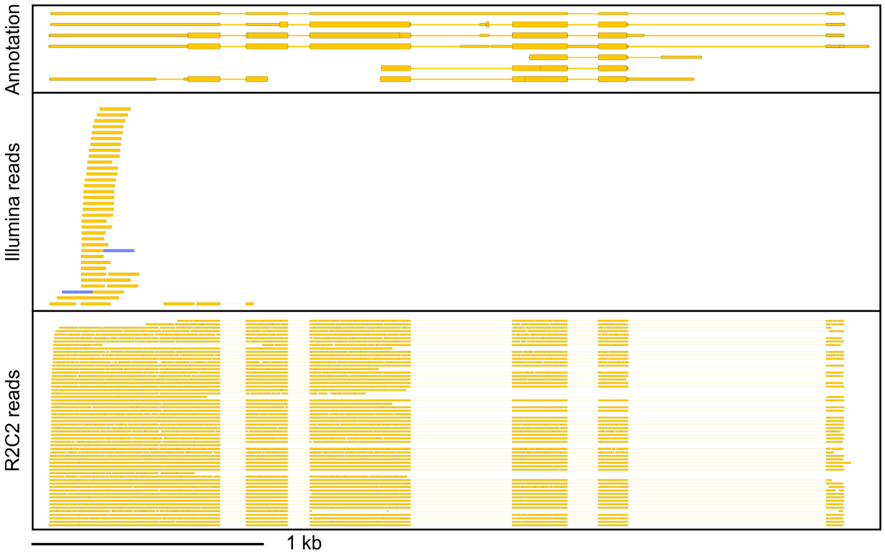

Supplementary Figure S7: R2C2 reads sequence 10X full-length cDNA transcripts. Genome Browser shots of ACTB. Genome annotation is shown on top and Illumina reads (center) R2C2 reads (bottom) aligning to the locus are shown below. Both Illumina and R2C2 read alignments were randomly subsampled to 60 reads. The directionality of features is indicated by color (“top strand”=blue, “bottom strand”=yellow). Data for replicate 1 are shown.

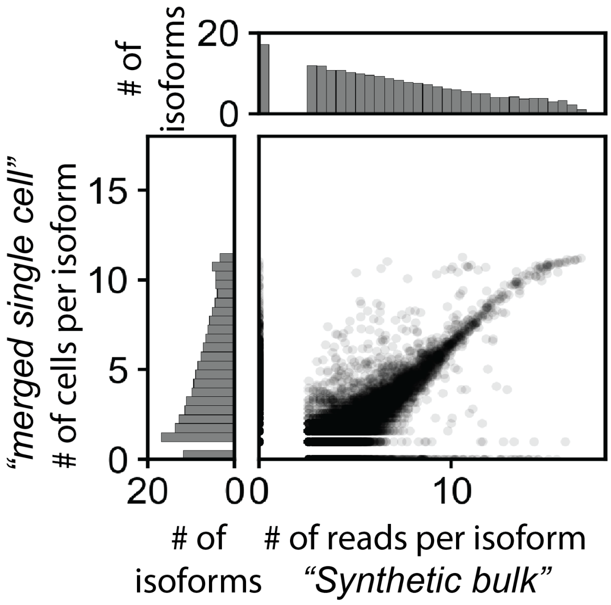

Supplementary Figure S8: Comparing isoform abundance between "synthetic bulk" and "merged single cell" approaches. Reads per isoform and cells per isoform in the respective approaches are shown as a scatter plot with associated marginal distributions. All values are log2(value+1) converted.

Replicate 1	Basecalled raw reads	Median raw read len	R2C2 Consensus reads
PromethION run 1	14,321,713	3601	8,588,694 (59.97%)
MinION run 1	2,476,880	4515	2,143,624 (86.55%)
PromethION run 2	12,069,611	3767	7,140,994 (59.17%)
MinION run 2	660,975	4074	547,384 (82.81%)
**Total**	**29,529,179**		**18,420,696 (62.38%)**

Replicate 2	Basecalled raw reads	Median raw read len	R2C2 Consensus reads
PromethION run 1	21,660,888	2746	13,603,254 (62.80%)
MinION run 1	4,865,719	2980	3,520,670 (72.36%)
**Total**	**26,526,607**		**17,123,924 (64.55%)**

Table S1: Oxford Nanopore Technologies sequencing run and read numbers. Values in parentheses indicate the percentage of raw reads being successfully converted into consensus reads. Note that R2C2 Consensus read numbers indicate consensus reads prior to post-processing. R2C2 Consensus reads after post-processing are given in Table 1.

Replicate 1

R2C2 reads combined into merged read	Number of merged reads	Median accuracy
2	915918	99.3%
3	360802	99.5%
4	158528	99.6%
5	82406	99.6%
6	41894	99.6%
7	23435	99.6%
8	15284	99.6%
9	9572	99.6%
10+	17890	99.5%

Replicate 2

R2C2 reads combined into merged read	Number of merged reads	Median accuracy
2	736417	99.2%
3	268047	99.5%
4	116908	99.6%
5	63638	99.6%
6	36752	99.6%
7	23558	99.6%
8	16875	99.6%
9	12238	99.6%
10+	40122	99.6%

Table S2: UMIs allow the merging of R2C2 reads originating from the same cDNA molecule.
