# Supplementary material for: Single-cell isoform analysis in human immune cells
Source: Genome Biol. 2022 Feb 7;23:47. doi: 10.1186/s13059-022-02615-z (PMC8819920; doi:10.1186/s13059-022-02615-z)
Supplement: Supplementary file 4 — Additional file 4. Peer review history. [file 13059_2022_2615_MOESM4_ESM.docx]

**Review history**

**First round of review**

**Reviewer 1**

Volden et al present a method based on previous published work in which they profile thousands of cells using ONT long-read single-cell sequencing. The main difference between their previous R2C2 manuscript (PNAS 2018) and the current one is that they switch from using smartseq2 to droplet based sequencing. The strengths of the manuscript are that it is largely well-written (with some important information missing though), the software approach looks to be sound and the methodology in the manuscript is of good quality (Its really quite an elegant approach). However, I have a few reservations with regards to the way the data is introduced and discussed. Specifically, I found it difficult to understand the difference between this manuscript and your previously published R2C2 method, it required me to read the PNAS paper. Ideally the manuscript should stand on its own and the novelty of the manuscript should be emphasised and defined better.Furthermore, I have reservations regarding the UMI assignment accuracy. While 67% of UMI matches between Illumina and Nanopore are good, this is still very low to be useful for accurate differential expression. The implications of this high error rate should be better described in the discussion.

I include my specific comments below:


Title:
1. I find the title of the manuscript slightly confusing. The inclusion of the words "Highly multiplexed" suggested to me that you have developed a novel method for multiplexing your samples, but if I'm not mistaken the R2C2 does not provide a method for multiplexing, but a method for error correcting barcode sequences.

2. Also, 10X genomics is a company name, the name for their sequencing platform is the 10X chromium.

3. A more applicable title highlighting the strengths of the manuscript would be beneficial for readers to interpret the novelty of the work.

Introduction:
4. You are missing a reference to the InDrops method! I see this a lot, but the InDrops manuscript is still a vitally important ref to be included.

5. I feel that references to the R2C2 method having been already published in PNAS in 2018 is glossed over within the introduction. I had to read carefully the PNAS paper to work out what the main differences were with the methodology implemented within this manuscript. If im not mistaken the main difference is its application to droplet based single-cell sequencing (10X chromium vs smart-seq2) and further refinement of the computational tools to make it applicable to TCR and Ig transcripts. Specific reference to the PNAS paper and its contrast to the current manuscript should be strengthened.

6. Missing references to other approaches for ONT sequencing should be included:
https://www.biorxiv.org/content/10.1101/2020.07.27.222349v1
https://www.nature.com/articles/nbt.4259
https://www.nature.com/articles/s41587-021-00965-w

Results:
7. This recent publication has shown that there are a lot of artefacts (PCR and sequencing chimeric reads) generated for both ONT and PacBio sequencing https://www.nature.com/articles/s41592-020-01041-y?proof=t . How does your R2C2 method deal with chimeric artefacts generated during library prep? What are the percentage of reads you are detecting that are artefacts? This could be addressed by performing a mouse and human mixing experiment and looking for the presence of fusion events between different reads. Given the extra length of your reads, this would be critical to understand the frequency of fusion events generated by your approach.

8. What is missing from the manuscript is any reference to the coverage of each UMI in a single read. A bar graph showing the frequency of numbers of UMIs per read should be added as a supplementary figure.

9. I have reservations regarding the accuracy of your UMI assignment. 67% UMIs similarity within the R2C2 data and gold standard Illumina data seems very low to accurately deduplicate UMIs at the single-cell level. While a result is a result, this should be discussed further with respect to the limitations and advantages of your R2C2 method. Can your method be used to accurately perform differential expression between different treatments/conditions? If it can then this should be demonstrated, but from the numbers I see quoted in the manuscript I very much doubt that this would be possible. While this isn't a problem in itself as you could still use your method for differential isoform usage within a samples, this should be discussed.

10. Lengthening the UMI would likely give better UMI assignment. Can the authors speculate/model what the best length of UMI would be to increase the UMI assignment rate to greater than 90%?

11. At line 54 you state "However, although their RNA molecule of origin cannot be unambiguously identified, R2C2 reads containing UMIs with sequencing errors are still highly valuable for downstream analysis". Given the significant number of errors that you see within your UMIs, you should elaborate on why the UMIs would be valuable given the high number of errors. Im not convinced that UMIs would be useful given their error rate. This "highly valuable for downstream analysis" needs to be clarified.

12. The manuscript would benefit from having a dot plot with the number of genes detected per cell for Illumina vs R2C2 for every cell.

13. Further quality control plots showing the number of UMI counts per cell, number of genes per cell and the relationship between the number of features and number of counts (i.e. all standard Seurat QC plots) should be shown in supplementary with the Illumina and R2C2 method presented side by side.


Discussion:
14. Your first line of the discussion states "Here, we present a method to analyze highly-multiplexed full-length single-cell transcriptomes that does not require short-read sequencing." But this makes the reader think that this is the novelty of the paper and its not, as the method was originally published in 2018 by the current group. The novel aspect is the application of R2C2 to droplet based sequencing. Throughout the manuscript the main novelty of the paper is muddled and buried and should be sharpened so the reader can better understand the differences between the PNAS paper and the current manuscript.

15. The authors state that they sequence 9 million unique transcripts from 3000 PBMCs. Looking at Table 2, which I'm assuming this is where this number comes from, there are fewer than 9 million reads (8,886,966). The authors should change this sentence to "The authors state that they sequence 8.8 million reads from 3000 PBMCs". Numbers should not be inflated to make the method look more impressive. For this type of publication, numbers really matter and help the reader interpret and place your method into context with other methods.

16. Furthermore, only having 8.8 million reads means that there are only ~3,000 reads sequenced per cell. This is very low. For differential gene expression, 10X recommend at least 20,000 reads per cell. Given the increased complexity (number of transcripts > number of genes) within the counts data for transcripts, it is likely that even more reads would be required to perform differential expression. In order for this method to become accurate at performing differential transcript expression, the authors either need to substantially increase the sequencing that they perform or state clearly within their manuscript that this is a limitation.

17. Given the significant PCR amplification and sequencing errors that can occur with ONT library prep and sequencing, UMIs have been shown to be very important for cross platform demultiplexing of singe-cell reads. Without the inclusion of a UMI sequence you see huge amplification biases, which create significant problems for downstream differential expression analysis. Your results only show a 67% UMI match with gold-standard Illumina sequencing data. You gloss over this in the results table and dont really discuss this properly. Accurate assignment of UMIs is critical for differential gene expression, for Nanopore they are critical even for bulk level analyses (https://www.nature.com/articles/s41467-020-19687-9 and https://www.nature.com/articles/s41592-020-01041-y?proof=t). When the authors say at line 54 "However, although their RNA molecule of origin cannot be unambiguously identified, R2C2 reads containing UMIs with sequencing errors are still highly valuable for downstream analysis", are they stating that with the R2C2 method is not suitable for differential gene expression, but merely only suitable for evaluating isoform analysis? This to me would indicate that your method is not suitable for performing counts related differential expression. If you do not have a high UMI assignment then you will artificially increase your transcript counts per cell/cluster.

18. The R2C2 method and the low UMI calling rate should be discussed more and possible limitations of when their method should and shouldn't be used should be highlighted.

19. The method is focused on ONT sequencing. PacBio sequencing would offer similar error rates to Illumina sequencing. Further context on the advantages/disadvantages should be elaborated upon on alternative sequencing approaches.

20. Why do you get such a high barcode agreement but a low UMI agreement? Is this because of the lower complexity of barcode sequences vs UMI sequences? If so this should be discussed.

**Reviewer 2**

In this work, Volden and Vollmers describe application of their R2C2 method to single cell sequencing using the 10X platform to measure transcript isoforms. Because the novelty of this approach compared to other single-cell long-read approaches is the ability to depart from Illumina, more information needs to be given in terms of how the single-cell analysis compares between Illumina and R2C2. The ability of this method to demultiplex a single-cell library is convincing; however, the limitations introduced by a lower sequencing depth with Nanopore need to be better addressed.

Specific Comments:
1. Relevant to discuss advantages over other papers purporting to use nanopore on 10X - e.g. sicelore (https://www.nature.com/articles/s41467-020-17800-6) or rage-seq (https://www.nature.com/articles/s41467-019-11049-4)
2. In Figure 1B, regarding the barcode rank plots for Illumina and R2C2: Are the UMIs/cell lower in R2C2 because of lower read output with ONT vs Illumina or is this from an inability to demultiplex the reads? I would assume the former, but it would be informative to clarify where you think this is coming from. Normalizing this versus coverage is probably also informative to get a good sense for how numbers of reads play in versus demultiplexing. Generally having an estimate for numbers of reads needed to achieve sufficient coverage vs numbers of cells would be useful.
3. In Table 1, regarding the number of Nanopore reads as they are processed in the C3POa pipeline: What is accounting for the major loss from basecalled reads to post-processed reads? Some discussion on this point is warranted.
4. For Figure 3, with regard to the comparative tSNE and feature expression plots between Illumina and R2C2:
1. Is the single-cell analysis for Illumina restricted to cells that were also found with R2C2? The number of cells looks similar, but with Illumina's higher sequencing depth I would expect greater cell number vs R2C2
2. It is likely that there are subtypes of cells within the T-cell group. Is R2C2 powerful enough to resolve these subtypes? Even if not, it would be interesting to see what resolution can be achieved with R2C2 with regard to more fine grained cell types, which are increasingly harder to identify with low sequencing depth.
5. With regard to the parameters chosen for single cell analysis: Why is there no lower limit for nCount_RNA? I understand that the lower sequencing output from Nanopore would result in lower UMIs/cell but I would be interested to see what R2C2 clustering would look like with more stringent cell inclusion criteria. Perhaps limiting analysis to cells that have higher UMIs/cell would result in better cell-type resolution, and the resulting detriments to cell number could be compensated for by merging multiple datasets.
6. With the Mandalorion pipeline, 5' end of transcript is determined with a custom leniency window for annotated TSS. For novel isoforms that are identified to have alternative TSS like AIF3 (Fig3b), it would be good to validate with CAGE-seq datasets. It can be useful also to include a plot for the read length distribution, and provide some metrics on all isoforms identified from Mandalorion. Especially thinking about what % of reads are in annotated TSS vs. unannotated because of the documented 3' bias for any poly-A based reverse transcription.
7. How does the mandalorian transcript isoform calling compare to other isoform tools i.e. stringtie2, FLAIR, LIQA, TALON?
8. By merging isoforms expressed by different cells, the author effectively increases the sequencing depth to identify more candidate isoforms, especially isoforms that are lowly expressed in single cells. In this study, a minimum of 2 reads is required to identify an isoform, which seems to be too few. What's the rationale behind this? In other words, how do you validate that these identified novel isoforms are real? The criteria used to identify and/or quantify novel isoforms could be better documented in the method section.
9. This low threshold could explain why 1% threshold for bulk analysis doesn't pick up the isoform. If it's present in merged single cells, is it just that it pops in enough cells to met the threshold (more than one cell) but not enough for bulk (1%)? Can the authors plot the distribution of how many cells had an isoform and how many reads per isoform?
10. Also from Figure 4 it looks like a lot of the introns found from merged single cell are just incompletely spliced? What % of the isoforms present in merged single cell but not pseudobulk were just intron retention events (Figure 4D)?
11. Further have the authors investigated if there is any bias for (or against) associated with the (much longer) intron retained isoforms from R2C2? In previous work the authors demonstrated no size bias with isoforms as long as 2.5kb, but human introns are quite long and could introduce bias associated with PCR or circularization - measuring the number of intron retained isoforms from illumina data (limited though it may be) versus R2C2 might be illustrative.
12. To get a sense of how much deeper sequencing would benefit the experiment, it may be useful to do a rareificaiton analysis - at least for the merged single cells and/or the pseudobulk analysis.
13. For Figure 5, are more accurate R2C2 reads necessary for capturing adaptive immune receptor (AIR) transcripts reliably? If accuracy is not the major factor, I am concerned that R2C2 can be of lower throughput than regular single cell nanopore sequencing, and thus limit the power of characterizing the pairing of AIR transcripts, where number of B cells (179 cells) are significantly less than that of T cells (2,199 cells).

**Authors’ response to reviewers**

Reviewer #1: Volden et al present a method based on previous published work in which they profile thousands of cells using ONT long-read single-cell sequencing. The main difference between their previous R2C2 manuscript (PNAS 2018) and the current one is that they switch from using smartseq2 to droplet based sequencing. The strengths of the manuscript are that it is largely well-written (with some important information missing though), the software approach looks to be sound and the methodology in the manuscript is of good quality (Its really quite an elegant approach).

Thank you!

However, I have a few reservations with regards to the way the data is introduced and discussed. Specifically, I found it difficult to understand the difference between this manuscript and your previously published R2C2 method, it required me to read the PNAS paper. Ideally the manuscript should stand on its own and the novelty of the manuscript should be emphasised and defined better.Furthermore, I have

reservations regarding the UMI assignment accuracy. While 67% of UMI matches between Illumina and Nanopore are good, this is still very low to be useful for accurate differential expression. The implications of this high error rate should be better described in the discussion.

I include my specific comments below:

Title:

1. I find the title of the manuscript slightly confusing. The inclusion of the words "Highly multiplexed" suggested to me that you have developed a novel method for multiplexing your samples, but if I'm not mistaken the R2C2 does not provide a method for multiplexing, but a method for error correcting barcode sequences.

That’s a fair point. In response to this and the next concern we proposed a different title: “*Single-cell isoform analysis in human immune cells*”

2. Also, 10X genomics is a company name, the name for their sequencing platform is the 10X chromium.

See above

3. A more applicable title highlighting the strengths of the manuscript would be beneficial for readers to interpret the novelty of the work.

Maybe make the isoform analysis the focus? That’s the mainly novel part of this anyway. So possibly something like

*See above*

Introduction:

4. You are missing a reference to the InDrops method! I see this a lot, but the InDrops manuscript is still a vitally important ref to be included.

We corrected this oversight

5. I feel that references to the R2C2 method having been already published in PNAS in 2018 is glossed over within the introduction. I had to read carefully the PNAS paper to work out what the main differences were with the methodology implemented within this manuscript. If im not mistaken the main difference is its application to droplet based single-cell sequencing (10X chromium vs smart-seq2) and further refinement of the computational tools to make it applicable to TCR and Ig transcripts. Specific reference to the PNAS paper and its contrast to the current manuscript should be strengthened.

This is correct. To make it more clear that this is only a modification of the R2C2 method paired with new computational tools, the last paragraph of the introduction now reads like this:

*In this manuscript we demonstrate that this combination of high throughput and accuracy is sufficient for the Illumina short-read independent analysis of highly multiplexed single-cell cDNA pools generated by the 10x Genomics Chromium controller. We independently analyzed two pools containing the cDNA molecules with a combined ~3000 human Peripheral Blood Mononuclear Cells (PBMCs) with Illumina and the established R2C2*[*(Volden et al. 2018)*](https://paperpile.com/c/ibfBSr/zHx8) *(ONT) workflows.* ***To this end, we modified the R2C2 workflow to be compatible with cDNA generated by the 10x Chromium controller and implemented new computational tools to identify 10x molecular and cellular identifiers.*** *By merging reads based on the molecular identifiers and demultiplexing reads based on their cellular identifiers we showed that the R2C2 approach identifies the same cellular identifiers in the cDNA pools and generates comparable single-cell gene expression profiles and cell type clusters as Illumina-based sequencing. In addition, and in contrast to Illumina data, R2C2 data also allow the determination of cell type specific and single-cell isoform-level transcriptomes. Finally, we developed a set of computational tools that allowed us to process R2C2 data to resolve and pair full-length adaptive immune receptors (AIR) transcripts in the B and T cell subpopulations of our PBMC sample which currently requires specialized library preparation methods and sequencing approaches.*

6. Missing references to other approaches for ONT sequencing should be included:

<https://www.biorxiv.org/content/10.1101/2020.07.27.222349v1> <https://www.nature.com/articles/nbt.4259> <https://www.nature.com/articles/s41587-021-00965-w>

We added the missing references. Results:

7. This recent publication has shown that there are a lot of artefacts (PCR and sequencing chimeric reads) generated for both ONT and PacBio sequencing <https://www.nature.com/articles/s41592-020-01041-y?proof=t>. How does your R2C2 method deal with chimeric artefacts generated during library prep? What are the percentage of reads you are detecting that are artefacts? This could be addressed by performing a mouse and human mixing experiment and looking for the presence of fusion events between different reads. Given the extra length of your reads, this would be critical to understand the frequency of fusion events generated by your approach.

While performing a human/mouse mixture experiment would be informative, I believe we can derive the same information from the data we have already produced. PCR artefacts, library prep, or analysis artefacts would all produce chimeric reads even if derived from only human cells. In order to quantify this, we calculated the percentage of chimeric read alignments across all reads produced for this study. By parsing the bit flag present in the sam output of minimap2, we determined the chimeric reads to be 1.7% of the dataset. This is certainly not zero, and honestly more than we were expecting, but it is lower than the article reports for PacBio CCS reads (1.9%) which are the closest comparison in terms of number and accuracy to R2C2 reads. Ultimately chimeric read alignments are filtered by Mandalorion so they shouldn’t affect isoform identification and quantification.

8. What is missing from the manuscript is any reference to the coverage of each UMI in a single read. A bar graph showing the frequency of numbers of UMIs per read should be added as a supplementary figure.

In response to this and other points, we have now added more information on the R2C2 reads including how often each molecule (UMI) was covered by ONT raw subreads:

*“We generated 11,564,494 and 10,661,139 R2C2 consensus reads with average subread coverage of* ***3.04 and 1.89*** *for replicate 1 and replicate 2 respectively. We then merged 3.3% (Rep1) and 6.5% (Rep2) of these R2C2 consensus because their Splint-UMI identified them as originating from the circularization of the same cDNA molecule. Second, we merged 46.3% and 46.1% of these Splint-UMI merged R2C2 consensus reads in replicate 1 and replicate 2, respectively, because their 10X-UMI identified them as originating from the same RNA molecule. Across both replicates this sequential merging process resulted in 14,822,072 Splint/10X-UMI merged R2C2 consensus reads (Table S2) with an average subread coverage of* ***3.73*** *(Fig. S1), an average sequence length of 1358 bp (Fig. S2) and median sequence accuracy of 98.0%.”*

We also added the requested subread coverage bar graph pre and post Splint and UMI based merging of R2C2 reads as Fig. S1)

**Supplementary Figure S1: Subread coverage per molecule.** Top: subread coverage per molecule before merging splint and 10x UMIs. Bottom: subread coverage per molecule after merging splint and 10x UMIs. Molecules with 0 subreads are assembled from overlapping incomplete subreads.

9. I have reservations regarding the accuracy of your UMI assignment. 67% UMIs similarity within the R2C2 data and gold standard Illumina data seems very low to accurately deduplicate UMIs at the single-cell level. While a result is a result, this should be discussed further with respect to the limitations and advantages of your R2C2 method. Can your method be used to accurately perform differential expression between different treatments/conditions? If it can then this should be demonstrated, but from the numbers I see quoted in the manuscript I very much doubt that this would be possible. While this isn't a problem in itself as you could still use your method for differential isoform usage within a samples, this should be discussed.

To address this and other points we added following language to the discussion:

*“As of now, potential users of this technology will have to decide whether to include these unmatched molecules in their downstream analysis. While these molecules are likely to help define isoforms by increasing read depth, they might also distort gene and isoform quantification. An increase in accuracy would make future demultiplexing and UMI merging steps more efficient and hopefully increase UMI matching rate to a point where this decision does not have to be made and different treatment and conditions can be safely compared across experiments.”*

10. Lengthening the UMI would likely give better UMI assignment. Can the authors speculate/model what the best length of UMI would be to increase the UMI assignment rate to greater than 90%?

There are now approaches which use modified cellular identifiers consisting of dimers which seem to be doing very well. (<https://www.nature.com/articles/s41587-021-00965-w>). While simulating this might be possible, it also would be based on a lot of arbitrary assumptions. More importantly however, the whole point of this manuscript is the ability to use the 10x platform without modifications. If the underlying molecular biology has to be changed to accomodate a long-read method, I think the amount of users for this long-read method will be very low. If we were to redo this experiment today and wanted to improve UMI assignment, we would sequence longer R2C2 DNA to generate more passes per molecule and use a better basecaller (i.e. guppy5). Doing this, we have already achieved read accuracy pre UMI-merging of 99.5% as opposed to ~98% in this study post UMI merging. We are basically already achieving Illumina-level read accuracies with this method so UMI assignment will become much less of an issue going forward.

11. At line 54 you state "However, although their RNA molecule of origin cannot be unambiguously identified, R2C2 reads containing UMIs with sequencing errors are still highly valuable for downstream analysis". Given the significant number of errors that you see within your UMIs, you should elaborate on why the UMIs would be valuable given the high number of errors. Im not convinced that UMIs would be useful given their error rate. This "highly valuable for downstream analysis" needs to be clarified.

It is correct that the UMIs themselves are not useful. What we meant to express here is that the reads themselves and their alignments carry information on isoforms. We have clarified the paragraph to now read like this:

*“Although their RNA molecule of origin cannot be unambiguously identified, we chose to include these R2C2 reads in our downstream analysis, thereby valuing the extra information they might contain for isoform identification over their potential to distort the quantification of gene and isoform expression.”*

12. The manuscript would benefit from having a dot plot with the number of genes detected per cell for Illumina vs R2C2 for every cell.

We have now added this plot as Fig. S3. genes detected per cell for Illumina vs R2C2 show an Pearson’s r of 0.94.

**Supplementary Figure S4: Genes per cell in R2C2 and Illumina.** The number of genes expressed by each cell as determined by R2C2 and Illumina is shown as a scatter plot.

13. Further quality control plots showing the number of UMI counts per cell, number of genes per cell and the relationship between the number of features and number of counts (i.e. all standard Seurat QC plots) should be shown in supplementary with the Illumina and R2C2 method presented side by side.

The manuscript already shows UMI counts per cell in Figure 1B, we have added a “genes per cell” plot as Fig. S4 as requested by the previous point. We now also added a “number of features and number of counts” plot as Fig. S5.

**Supplementary Figure S5: Number of molecules vs. number of genes per cell**. The number of molecules (nCount_RNA) was plotted against the number of genes (nFeature_RNA) for each cell as scatter plots for both RNA and Illumina datasets. Pearson’s r values are shown on top of each plot.

Figs. S4 and S5 are now mentioned in the manuscript here:

*We then clustered R2C2 and Illumina datasets independently using the Seurat analysis package* [*[34]*](https://paperpile.com/c/ibfBSr/kgMNn)*. R2C2 and Illumina datasets both generated highly similar library metrics as determined by Seurat, i.e. genes (nFeatures) and molecules (nCount_RNA) per cell (Fig. S4 and S5).*

Discussion:

14. Your first line of the discussion states "Here, we present a method to analyze highly-multiplexed full-length single-cell transcriptomes that does not require short-read

sequencing." But this makes the reader think that this is the novelty of the paper and its not, as the method was originally published in 2018 by the current group. The novel aspect is the application of R2C2 to droplet based sequencing. Throughout the manuscript the main novelty of the paper is muddled and buried and should be sharpened so the reader can better understand the differences between the PNAS paper and the current manuscript.

To address this by now opening the discussion with:

*“Here, we present modified molecular biology workflows and new computational tools that make it possible to apply the R2C2 method to full-length single-cell cDNA pools generated by the droplet-based 10x Genomics Chromium controller.”*

15. The authors state that they sequence 9 million unique transcripts from 3000 PBMCs. Looking at Table 2, which I'm assuming this is where this number comes from, there are fewer than 9 million reads (8,886,966). The authors should change this sentence to "The authors state that they sequence 8.8 million reads from 3000 PBMCs". Numbers should not be inflated to make the method look more impressive. For this type of publication, numbers really matter and help the reader interpret and place your method into context with other methods.

We agree with the reviewer that there is no need to overstate the numbers and we should have gone with the more accurate read numbers to begin with.

While the older version of the manuscript indeed dealt with ~8.8 million reads, updating the basecaller and C3POa versions we actually produced over 12 million R2C2 reads (12,038,521) in this iteration of the manuscript. So, at the points in the manuscript where 9 million reads were mentioned the newer version now states 12 million which I think is appropriate.

I believe this mixup has to do with different versions of the manuscript that were uploaded to biorxiv and imported into the Genome Biology online system. There certainly are some issues because the abstract seems to be from the older version for example. We should have been more careful when transferring the manuscript. This is definitely on us. Apologies for that.

16. Furthermore, only having 8.8 million reads means that there are only ~3,000 reads sequenced per cell. This is very low. For differential gene expression, 10X recommends at least 20,000 reads per cell. Given the increased complexity (number of transcripts > number of genes) within the counts data for transcripts, it is likely that even more reads would be required to perform differential expression. In order for this method to become accurate at performing differential transcript expression, the authors either need to substantially increase the sequencing that they perform or state clearly within their manuscript that this is a limitation.

We agree that this is a limitation and that especially differential isoform expression analysis is likely to be limited to medium to highly expressed genes. We are now writing in the first paragraph of the discussion:

*“We sequenced these libraries and demultiplexed the resulting data to produce over 12 million unique transcript molecules generated from ~3000 PBMCs. This amounted to ~4000 R2C2 reads per cell as opposed to the 20,000 Illumina reads 10x Genomics recommends. At this coverage, low expressed genes are likely to be excluded from gene and isoform analysis. We nonetheless used these single cell data to determine...”*

17. Given the significant PCR amplification and sequencing errors that can occur with ONT library prep and sequencing, UMIs have been shown to be very important for cross platform demultiplexing of singe-cell reads. Without the inclusion of a UMI sequence you see huge

amplification biases, which create significant problems for downstream differential expression analysis. Your results only show a 67% UMI match with gold-standard Illumina sequencing data. You gloss over this in the results table and dont really discuss this properly. Accurate assignment of UMIs is critical for differential gene expression, for Nanopore they are critical even for bulk level analyses (<https://www.nature.com/articles/s41467-020-19687-9>and <https://www.nature.com/articles/s41592-020-01041-y?proof=t>). When the authors say at line 54 "However, although their RNA molecule of origin cannot be unambiguously identified, R2C2 reads containing UMIs with sequencing errors are still highly valuable for

downstream analysis", are they stating that with the R2C2 method is not suitable for differential gene expression, but merely only suitable for evaluating isoform analysis? This to me would indicate that your method is not suitable for performing counts related differential expression. If you do not have a high UMI assignment then you will artificially increase your transcript counts per cell/cluster.

As mentioned above, we now explain that we include these reads in downstream analysis at the danger of potentially distorting the gene and isoform expression.

*“Although their RNA molecule of origin cannot be unambiguously identified, we chose to include these R2C2 reads in our downstream analysis, thereby valuing the extra information they might contain for isoform identification over their potential to distort the quantification of gene and isoform expression.”*

Importantly, the inclusion of these reads does not distort gene expression measurements to a point where it changes cell cluster assignments as opposed to Illumina sequencing. As for isoform differential expression analysis, we believe that our chi-squared test is already very conservative by only detecting only 74 genes with potentially differentially expressed isoforms between cell types (all of which we have looked at individually).

18. The R2C2 method and the low UMI calling rate should be discussed more and possible limitations of when their method should and shouldn't be used should be highlighted.

We are now highlighting the trade-off of including molecules with unmatched UMIs in downstream analysis and that this is ultimately up to the individual scientist and use-case.

*“In the current study, with about 4000 R2C2 reads per cell, we captured about 67% of the molecules present in an exhaustively sequenced Illumina dataset of the same cDNA. This was sufficient to cluster cell types and generate single-cell transcriptomes. As of now, potential users of this technology will have to decide whether to include these unmatched molecules in their downstream analysis. While these molecules are likely to help define isoforms by increasing read depth, they might also distort gene and isoform quantification. An increase in accuracy would make future demultiplexing and UMI merging steps more efficient and hopefully increase UMI matching rate to a point where this decision does not have to be made.”*

19. The method is focused on ONT sequencing. PacBio sequencing would offer similar error rates to Illumina sequencing. Further context on the advantages/disadvantages should be elaborated upon on alternative sequencing approaches.

We have now added a sentence and some PacBio citations to the discussion indicating that PacBio is still more accurate than R2C2 (at least in this study):

*“While our demultiplexing strategy can handle sequencing errors (see Methods), at 98% accuracy it still only manages to demultiplex ~81% of R2C2 reads, which is better than previously published approaches, but not ideal* [*[24,30]*](https://paperpile.com/c/ibfBSr/BZD4x%2BC34yD)*. Increasing accuracy to the level of PacBio Iso-Seq[[23,25,40]](https://paperpile.com/c/ibfBSr/hsyjV%2Bqplu%2BqrKv) could increase this number significantly. Paired with the higher throughput we can achieve by optimizing raw read to consensus read conversion as we have previously shown* [*[41]*](https://paperpile.com/c/ibfBSr/XE2z)*, future experiments could only retain UMIs which were observed more than once, similar to how we analyze Illumina data (see Methods). “*

20. Why do you get such a high barcode agreement but a low UMI agreement? Is this because of the lower complexity of barcode sequences vs UMI sequences? If so this should be discussed.

Yes, barcode diversity is lower and in a first step, we define which cellular barcodes are the most common in the dataset and only compare reads to those. We have added the following text to the discussion:

*“The demultiplexing method we developed generates a pre-filtered list of the most common barcodes in a cDNA pool and then compares each R2C2 read’s cellular barcode to this list. This is a more efficient and straightforward approach than comparing molecular barcodes across all R2C2 reads. Further, our demultiplexing strategy can handle sequencing errors (see Methods), yet, at 98% read-accuracy, it still only manages to demultiplex ~81% of R2C2 reads. This is better than previously published approaches, but not ideal* [*[24,30]*](https://paperpile.com/c/ibfBSr/BZD4x%2BC34yD)*.“*

Reviewer #2: In this work, Volden and Vollmers describe application of their R2C2 method to single cell sequencing using the 10X platform to measure transcript isoforms. Because the novelty of this approach compared to other single-cell long-read approaches is the ability to depart from Illumina, more information needs to be given in terms of how the single-cell analysis compares between Illumina and R2C2. The ability of this method to demultiplex a single-cell library is convincing; however, the limitations introduced by a lower sequencing depth with Nanopore need to be better addressed.

Specific Comments:

1. Relevant to discuss advantages over other papers purporting to use nanopore on 10X - e.g. sicelore (<https://www.nature.com/articles/s41467-020-17800-6>) or rage-seq (<https://www.nature.com/articles/s41467-019-11049-4>)

In short, sicelore requires Illumina reads while RAGE-seq is a targeted approach that only covers immune receptor transcripts. We now cite both papers to highlight those differences. The first in the introduction when pointing out tools that need Illumina reads for analysis and the second in the last paragraph of the discussion when highlighting that the characterization of BCR and TCR sequences by our approach does not require specialized molecular methods.

2. In Figure 1B, regarding the barcode rank plots for Illumina and R2C2: Are the UMIs/cell lower in R2C2 because of lower read output with ONT vs Illumina or is this from an inability to demultiplex the reads? I would assume the former, but it would be informative to clarify where you think this is coming from. Normalizing this versus coverage is probably also informative to get a good sense for how numbers of reads play in versus demultiplexing. Generally having an estimate for numbers of reads needed to achieve sufficient coverage vs numbers of cells would be useful.

We agree with the reviewer that UMIs/cell are likely lower due to the fact that we have less reads to begin with with R2C2. Based on read/UMI coverage we are fairly certain that the Illumina data was sequenced to exhaustion while the R2C2 dataset was not. We added the following text to the results section:

*“Consequently, the number of unique molecules assigned to each cell was similar between the datasets, although the exhaustively sequenced Illumina dataset contained more molecules per cell than the non-exhaustive R2C2 dataset”*

Further, Table 1 describes in detail where we are losing reads during R2C2 processing and should give future users of this method a good idea what read numbers to aim for to achieve a certain number of reads per cell. This would likely be a conservative estimate as we are losing less reads on the first step (raw reads to consensus reads) these days.

3. In Table 1, regarding the number of Nanopore reads as they are processed in the C3POa pipeline: What is accounting for the major loss from basecalled reads to post-processed reads? Some discussion on this point is warranted.

Thank you for bringing this up. In short, those were bad runs of short DNA. We don’t see this drop in current datasets. Just for reference, our latest PromethION runs, which we did at UC Davis genomics core for the LRGASP consortium, generated ~20 million raw reads that were 7kb long on average of which >90% converted to consensus reads.

The PromethION runs we did for this paper were done by ourselves at UCSC and were the first PromethION runs we ever did at a time where PromethION flow cells still had a lot more variability. We didn’t want to address this because it would look a lot like throwing ONT under the bus.

We have added the following sentence to the discussion that acknowledges the low conversion rate and cites the LRGASP preprint is associated with more recent R2C2 data generated on the ONT MinION and PromethION:

*“Paired with the higher throughput we can achieve by optimizing raw read to consensus read conversion as we have previously shown* [*[41]*](https://paperpile.com/c/ibfBSr/XE2z)*”*

4. For Figure 3, with regard to the comparative tSNE and feature expression plots between Illumina and R2C2:
   1. Is the single-cell analysis for Illumina restricted to cells that were also found with R2C2? The number of cells looks similar, but with Illumina's higher sequencing depth I would expect greater cell number vs R2C2

Both datasets were processed entirely independently. However, both were limited to ~1500 cells. Considering the sharp drop-off of UMIs per cell in both R2C2 and Illumina data around cell 1500, we think this cut-off represents the approximate number of cells processed in that particular 10x run.

2. It is likely that there are subtypes of cells within the T-cell group. Is R2C2 powerful enough to resolve these subtypes? Even if not, it would be interesting to see what resolution can be achieved with R2C2 with regard to more fine grained cell types, which are increasingly harder to identify with low sequencing depth.

With the current coverage the T-cell subpopulations don’t separate in either the Illumina or R2C2 datasets, despite the Illumina dataset being sequenced to exhaustion. If we just clustered the T cells by themselves the sub populations might separate. In any case a 10x molecule carries the same information for gene expression based clustering whether it is sequenced by an Illumina sequencer or R2C2 so at the same coverage, both technologies should be equally powerful for separating cell types based on gene expression.

5. With regard to the parameters chosen for single cell analysis: Why is there no lower limit for nCount_RNA? I understand that the lower sequencing output from Nanopore would result in lower UMIs/cell but I would be interested to see what R2C2 clustering would look like with more stringent cell inclusion criteria. Perhaps limiting analysis to cells that have higher UMIs/cell would result in better cell-type resolution, and the resulting detriments to cell number could be compensated for by merging multiple datasets.

There is no lower limit for nCount_RNA because we already filter cells that have low UMI counts when we demultiplex reads. For the clustering, the cells with the most reads were typically T-cells. So if we were to exclude cells with lower UMI counts, we would be filtering out a lot of B-cells and monocytes.

6. With the Mandalorion pipeline, 5' end of transcript is determined with a custom leniency window for annotated TSS. For novel isoforms that are identified to have alternative TSS like AIF3 (Fig3b), it would be good to validate with CAGE-seq datasets. It can be useful also to include a plot for the read length distribution, and provide some metrics on all isoforms identified from Mandalorion. Especially thinking about what % of reads are in annotated TSS vs. unannotated because of the documented 3' bias for any poly-A based reverse transcription.

R2C2 read length had an average of 1358. Isoforms defined by Mandalorion had an average length of 1511bp. We have included these distributions in the supplement as Fig. S2. This is on the shorter end but still comparable to bulk transcriptome analysis experiments.

**Supplementary Figure S2: R2C2 read and Mandalorion isoform length distribution.** Read and isoform length distributions (as determined by Mandalorion for the “synthetic bulk” dataset) were determined by the length of the fasta sequence entries in the respective files.

We also used SQANTI3 on the set of isoforms used for differential expression analysis (Synthetic bulk) to determine the number of isoforms whose TSS overlap either with refTSS regions which are based on CAGE and other types of information.

We found that

90.58% of the 8390 fullsplice-match (FSM),

87.54% of the 2906 novel in catalog (NIC),

80.04% of the 1904 novel not in catalog (NNC), and 80.6% of the 3810 incomplete splice match (ISM)

isoforms classified by SQANTI for the isoform set overlapped with a refTSS region.

Overall these very high percentages make us confident in our isoform calls. We have included a summary number, i.e. 87% of all isoform having refTSS support to the manuscript:

*“To this end, we composed an additional “synthetic bulk” sample using the R2C2 reads from all cells in the dataset. We then used Mandalorion to identify all isoforms present in this “synthetic bulk” sample. In total, Mandalorion identified 17,010 isoforms at an average length of 1511 bp (Fig. S4). Similar to the individual cell type isoform sets, the majority (66%) of isoforms in this synthetic bulk isoform set were classified by SQANTI to be either ‘full-splice-match’ or ‘novel-in-catalog’. Importantly, the TSSs of 87% of all isoforms in this set had refTSS support which gave us high confidence in their 5’ ends.”*

We have added a refTSS track to the AIF1 genome browser shot to show that the new TSS indeed has refTSS support.

7. How does the mandalorian transcript isoform calling compare to other isoform tools i.e. stringtie2, FLAIR, LIQA, TALON

We have internally compared Mandalorion to stringtie2 , scallop, IsoSeq3, and TALON on single replicate samples (TALON performs better when replicates are available). In our hands it outperforms all of them, at least when applied to SIRV spike-ins.

Stringtie2 (conservative preset ‘StC’): Power 0.78 Precision 0.87 Scallop(Sc): Power 0.39 Precision 0.13

TALON(T): Power 0.94 Precision 0.23

IsoSeq3(I): Power 0.58 Precision 0.17

IsoSeq3+cupcake(I+C): Power 0.59 Precision 0.53

Mandalorion(M): Power 0.86 Precision 0.98

Mandalorion shows the best Precision/Power combination in my opinion. We decided to not publish Mandalorion by ourselves and instead join the more comprehensive LRGASP consortium bake-off. We are excited to see whether this internal validation will be reproduced by the LRGASP consortium.

8. By merging isoforms expressed by different cells, the author effectively increases the sequencing depth to identify more candidate isoforms, especially isoforms that are lowly expressed in single cells. In this study, a minimum of 2 reads is required to identify an isoform, which seems to be too few. What's the rationale behind this? In other words, how do you validate that these identified novel isoforms are real? The criteria used to identify and/or quantify novel isoforms could be better documented in the method section.

I think any minimum read requirement is always an (hopefully) informed choice. We selected 2 reads because in this dataset that actually corresponds to two independent molecules. We then also did analysis on how many independent cells expressed a given isoform. So we ultimately looked at isoforms that had at least two reads in at least two cells. For “synthetic bulk” analysis we actually used the default minimum read number of 5 to be more stringent because we didn’t keep track of the cell-level information. The exact commands that we used to run Mandalorion are included in the methods section.

Validating novel isoforms is extremely hard because long-read sequencing is literally the only way of easily observing full-length isoforms. The only way to validate new isoforms would be to use a combination of targeted PCR, 5’ and 3’ RACE, Northern Blots, etc… Nothing that is even remotely possible for more than a handful of isoforms and even then an unreasonable amount of work.

9. This low threshold could explain why 1% threshold for bulk analysis doesn't pick up the isoform. If it's present in merged single cells, is it just that it pops in enough cells to met the threshold (more than one cell) but not enough for bulk (1%)? Can the authors plot the distribution of how many cells had an isoform and how many reads per isoform?

Yes, it is correct that we are simply dealing with two separate filtering cut-offs here but that is also by design. Merged single cell transcriptomes can provide extra confidence for low abundance isoforms by showing that they are expressed by two or more independent cells.

Overall, the number of cells expressing an isoform and the total number of reads associated with this isoform are quite well correlated at a Pearson’s r of 0.71. But, as expected, there were many more merged single cell isoforms (with more than 2 cells expressing it) that didn’t have any matching synthetic bulk isoform (22,815) than there were synthetic bulk isoforms that didn’t have a matching single cell isoform (3760). We generated the requested plot and included it as supplementary figure S8.

We now refer to this figure now in the results section:

*“By systematically comparing the ‘merged single cell’ and ‘synthetic bulk’ approaches we showed that the number of cells expressing an isoform in the ‘merged single cell’ approach and the number of reads associated with that isoform in the ‘synthetic bulk’ approach correlated well (Pearson’s r=0.71, Fig. S8)”*

**Supplementary Figure S8: Comparing isoform abundance between “synthetic bulk” and “merged single cell” approaches.**

Reads per isoform and cells per isoform in the respective approaches are shown as a scatter plot with associated marginal distributions. All values are log2(value+1) converted.

10. Also from Figure 4 it looks like a lot of the introns found from merged single cell are just incompletely spliced? What % of the isoforms present in merged single cell but not pseudobulk were just intron retention events (Figure 4D)?

This is a deceptively simple, but really very complex, question. We agree with the reviewers that there seems to be a lot of intron retention events. However defining whether an isoform is unique just because of an intron retention event requires in-depth annotation of an isoform. I don’t think there is a good tool for doing this well in an automated way. This would basically have to be done through manually curation which at the scale of this dataset is not feasible.

However, in order to address the concern we now mention that many of the single cell isoforms seem to incompletely spliced:

*“Interestingly, much of the single cell isoform diversity we observe seemed to be based on intron retention and/or be incompletely spliced transcripts and varied greatly between genes (Fig. 4A).”*

11. Further have the authors investigated if there is any bias for (or against) associated with the (much longer) intron retained isoforms from R2C2? In previous work the authors demonstrated no size bias with isoforms as long as 2.5kb, but human introns are quite long and could introduce bias associated with PCR or circularization - measuring the number of intron retained isoforms from illumina data (limited though it may be) versus R2C2 might be illustrative.

The most straightforward answer to this question is that if an intron is longer than the reads we are generating (limited mostly by PCR in this manuscript) then we won’t observe it ever being retained. We have now plotted the length distribution of reads as requested in a previous point in Fig. S2 showing an average length of ~1300 bp with very few reads longer than 4kb. So any intron above ~4kb is unlikely to ever be observed as retained. This is a limitation that inherent to any method based on amplifying full-length cDNA (Smart-seq, 10x, etc...), i.e. RT/PCR will always prefer shorter molecules and not amplify the longest molecules. The Illumina data will have this same problem because it simply fragments the same cDNA (and only looks at the 3’ ends).

12. To get a sense of how much deeper sequencing would benefit the experiment, it may be useful to do a rareificaiton analysis - at least for the merged single cells and/or the pseudobulk analysis.

Single cell experiments present a fairly unique case in genomics where the number of molecules in a pool is the true limiting factor, not the potential number of reads one can generate. In our case, we know from the Illumina analysis that about 5k transcript molecules were generated per cell in this study. As a rule of thumb, a coverage of 20k reads per cell is recommended by 10x Genomics. This should be more than enough to extract all possible information out of this experiment. So our goal for the future is to match this recommendation or more generally, sequence deep enough so that we can only retain molecules observed more than once as we describe in the discussion:

*“Paired with the higher throughput we can achieve by optimizing raw read to consensus read conversion as we have previously shown* [*[42]*](https://paperpile.com/c/ibfBSr/XE2z)*, future experiments could only retain UMIs which were observed more than once, similar to how we analyze Illumina data (see Methods).”*

For a few thousand cells, we could do this today but it was out of our reach when we generated this dataset in 2019.

An important and limiting consideration here is that while it would indeed be interesting to perform a rarefaction experiment, the version of Mandalorion we used for this manuscript takes about 3 days to complete for the pseudobulk experiment and about a week to make it through all the single cell data just once. Because of the design of the Mandalorion software, its behavior can’t be inferred for a subsample of reads, instead any rarefaction analysis would involve running Mandalorion many times over, basically non-stop for a few months. We believe this to be outside the scope of this manuscript.

13. For Figure 5, are more accurate R2C2 reads necessary for capturing adaptive immune receptor (AIR) transcripts reliably? If accuracy is not the major factor, I am concerned that R2C2 can be of lower throughput than regular single cell nanopore sequencing, and thus limit the power of characterizing the pairing of AIR transcripts, where number of B cells (179 cells) are significantly less than that of T cells (2,199 cells).

This is a good point and ultimately doesn’t have an answer that we could give. If enough regular nanopore reads are collected, their consensus shouldn’t be any worse than a consensus based on R2C2 reads. But, while ONT sequencing runs generate more regular nanopore reads than they do R2C2 reads (factor of 4-5) those reads are going to be lost at a higher frequency through demultiplexing, UMI matching, and alignment and are going to be much shorter on average. Standard cDNA sequencing on the ONT MinION has a massive length bias generating median read lengths ~500 bp. IGH transcripts can be up to 2 kb long meaning that they will very likely be sequenced very inefficiently. I would be excited to see this experiment done but don’t think it is on us to show that a different approach might be feasible for this application as this is no doubt going to require serious optimization and tool development.

**Second round of review**

**Reviewer 1**

The authors should be commended on their responses to the questions raised by myself. The manuscript now reads much better and a lot of the points have been addressed satisfactorily. However, I do have a final concern that needs to be addressed:

10. Lengthening the UMI would likely give better UMI assignment. Can the authors
speculate/model what the best length of UMI would be to increase the UMI assignment rate to
greater than 90%?
There are now approaches which use modified cellular identifiers consisting of dimers which
seem to be doing very well. (https://www.nature.com/articles/s41587-021-00965-w). While
simulating this might be possible, it also would be based on a lot of arbitrary assumptions. More
importantly however, the whole point of this manuscript is the ability to use the 10x platform
without modifications. If the underlying molecular biology has to be changed to accomodate a
long-read method, I think the amount of users for this long-read method will be very low. If we
were to redo this experiment today and wanted to improve UMI assignment, we would
sequence longer R2C2 DNA to generate more passes per molecule and use a better basecaller
(i.e. guppy5). Doing this, we have already achieved read accuracy pre UMI-merging of 99.5%
as opposed to ~98% in this study post UMI merging. We are basically already achieving
Illumina-level read accuracies with this method so UMI assignment will become much less of an
issue going forward.

Technology, especially single-cell technology changes considerably almost on a daily basis. This is also the case for the single-cell technology at 10X. The dimer manuscript you highlight looks like an attractive approach to handle UMI errors and I don't see a technical reason 10X or other single-cell sequencing providers couldn't adopt this approach in the future. Despite this no method is perfect, but I am still worried that your UMI correction approach lacks the necessary accuracy required to perform highly accurate quantitative differential expression. However, I think you have gone some way to highlighting this now in your manuscript. Therefore, given that I still have reservations regarding the UMI accuracy I think this should be emphasised further in the discussion of the manuscript. Specifically, highlighting that the method has potential pitfalls with regards to the UMI accuracy when ran on its own. I think you should highlight the use of dimers as an improved solution, however there are other approaches for correcting UMIs that may also provide more accuracy, such as combining with short read sequencing (https://www.nature.com/articles/s41467-020-17800-6).

For clarify, I'm not saying that there is anything wrong with your approach, as I really do like the method, but I think the reader should be aware of the pitfalls of the method and judge them appropriately for their applications.

**Reviewer 2**

The authors have done a good job overall responding to my comments, and I agree that the method and work has clear utility, but I still think they need to be clearer that R2C2 suffers from (even in the more recent LRGASP preprint they cite) a significant drop in yield in favor of the increased accuracy. Some detailed comments about this below.

3. The authors say that the read yield is better with more recent runs - but the LRGASP runs were not single cell samples - and the yield is still significantly lower than expected from linear cDNA runs? Looking at Table 7 in the LRGASP preprint cited R2C2 used 6 flowcells to achieve only 8M reads, compared to the 60M reads from *3* flowcells? This lower yield should be address head on in the paper, that there is a sacrifice of yield for accuracy. This is also compared to Table S1 in _this_ paper where the yield for both minION and promethion are relatively low. This is especially important in contrast to PacBio yield, which (one could argue) the same sacrifice for read number vs accuracy is made.

8. I'd argue that . . . . some of the isoforms can be validated by short-read sequencing confirming some of the novel reconstructions - i.e. that NIC transcripts have some reads which span that exon-exon boundaries. But this is fair enough, I think I'm more asking the % of transcripts which are "expected" which the authors discuss in response to Rev1 (comment 6)

11. Is it in fact so that the primary limitation of length is in the early PCR steps? It seems to me that circularization for R2C2 might also have some dependency on the size of the molecule - have the authors investigated this? Later (comment 13) the authors assert that cDNA sequencing has a length bias presumably this is from the PCR as direct cDNA shown in Chen et al (bioRxiv 2021) has a different median read length than cDNA-PCR. It's not clear to me why the authors don't observe the same size bias - is this just because their splint anchors to the strand-switching oligo? Examining Figure S2 their modal read length seems to be down around the same range, though Table S1 reports a high median "raw" read length, that doesn't mean their consensus read length is that long?

12. The authors assert here that there are 5000 molecules produced per cell (Figure 1), but pretty clearly from Figure 1B R2C2 does not capture the number of UMIs (per cell rank) that Illumina does? This is further confusing as it seems that there were *fewer* genes detected per cell in Illumina than R2C2 (Figure S4). It isn't clear that this is saturation level sequencing even for the Illumina data - Zhang et al (PMID: 30472192) found that more sequencing allows more gene detection.

Though I can accept the problem with mandalorian, I do think that a rarefication analysis _is_ within the scope, because it allows the reader to get a sense for _how deeply_ we need to sequence to detect isoforms. As nanopore sequencing (especially with R2C2, see comments above about Table 7) and PacBio sequencing for that matter both are relatively limited in numbers of reads/$ as compared to say Illumina (which admittedly only gives gene level information) - use of this method with experimental design requires a sense of the depth of sequencing needed. If not a rarefication analysis, then at least a discussion from other (perhaps bulk) data of guidelines for depth of sequencing needed per sample to achieve the numbers of cells needed.

**Authors’ response to reviewers**

Reviewer 1 Final Concern

Technology, especially single-cell technology changes considerably almost on a daily basis. This is also the case for the single-cell technology at 10X. The dimer manuscript you highlight looks like an attractive approach to handle UMI errors and I don't see a technical reason 10X or other single-cell sequencing providers couldn't adopt this approach in the future. Despite this no method is perfect, but I am still worried that your UMI correction approach lacks the necessary accuracy required to perform highly accurate quantitative differential expression. However, I think you have gone some way to highlighting this now in your manuscript. Therefore, given that I still have reservations regarding the UMI accuracy I think this should be emphasised further in the discussion of the manuscript. Specifically, highlighting that the method has potential pitfalls with regards to the UMI accuracy when ran on its own. I think you should highlight the use of dimers as an improved

solution, however there are other approaches for correcting UMIs that may also provide more accuracy, such as combining with short read sequencing (https://www.nature.com/articles/s41467-020-17800-6).

For clarify, I'm not saying that there is anything wrong with your approach, as I really do like the method, but I think the reader should be aware of the pitfalls of the method and judge them appropriately for their applications.

This is a fair point. We hope we sufficiently address it now by having added the following text to the discussion:

Beyond improving the R2C2 method itself, a tempting approach would of course be to use Illumina short-reads to aid the cell barcode and UMI sequence assignment [27]. Further, any error/indel prone long-read method could benefit from a redesign of cell barcode and UMI sequences present in the oligos used by the 10X Genomics workflow. A recent example of a long-read appropriate design [25] used homodimeric nucleoside phosphoramidite building blocks to synthesize cellular barcodes and UMI sequences composed of sequence dimers to improve demultiplexing and molecule assignments.

The question remains whether cell barcodes and UMI sequences can be improved for long-read sequencers with more subtle changes not requiring specialized oligo synthesis. Currently designed exclusively for short-read sequencers, both 16nt cell barcode and 10nt UMI sequence are present in the 10X oligodT primer directly adjacent to each other. Cell barcode and UMI sequence can therefore only be parsed from a sequencing read based on their sequence distance from the constant part (PCR handle) of the oligodT primer. This means that an indel in the cell barcode will also affect the UMI sequence next to it, thereby aggravating the consequences of the most common long-read sequencing error-type. This is made even more problematic because the UMI sequence is directly adjacent to the actual oligodT stretch of the oligodT primer - a long stretch of Ts which is notoriously hard for long-read sequencers to get right and will also likely affect the sequences adjacent to it.

We propose that future iterations of the oligodT primer contain spacer sequences of known length and sequence at defined positions between and within the cell barcodes and UMI sequences.

Instead of and oligodT primer with the following structure:

[PCR_handle]XXXXXXXXXXXXXXXXNNNNNNNNNNTTTTTTTTT

with X denoting variable bases of the cell barcode, N variable bases of the UMI sequence, and T actual T bases of the oligodT primer, we propose a oligodT primer structure as follows:

[PCR_handle]XXXXXATAXXXXXTATXXXXXXCTCNNNNNGAGNNNNNACATTTTTTTTT

Where the bolded A, T, C, and G bases create sequence spacers that can be used to easily parse cell barcodes and UMI sequences as well as immediately detect and mitigate indel errors. Because read positions with the exact same base in all sequenced molecules can be problematic for Illumina sequencers, there could be 4 different combinations of these spacers to make sure the read positions they occupy have a balanced base composition.

Reviewer 2 concerns:

3. The authors say that the read yield is better with more recent runs - but the LRGASP runs were not single cell samples - and the yield is still significantly lower than expected from linear cDNA runs? Looking at Table 7 in the LRGASP preprint cited R2C2 used 6 flowcells to achieve only 8M reads, compared to the 60M reads from *3* flowcells? This lower yield should be address head on in the paper, that there is a sacrifice of yield for accuracy. This is also compared to Table S1 in _this_ paper where the yield for both minION and promethion are relatively low. This is especially important in contrast to PacBio yield, which (one could argue) the same sacrifice for read number vs accuracy is made.

To highlight the fact that R2C2 sacrifices throughput for accuracy, we modified the paragraph introducing the R2C2 method to now read like this:

We have previously developed and applied the R2C2 approach which uses concatemeric consensus sequencing to improve ONT read accuracy from ~92% to >99% while still producing more than 2 million full-length cDNA sequences per MinION flow cell [19,20,29,30]. This increase in accuracy however comes with a decrease in read throughput as regular cDNA ONT runs can yield from 10-20 million reads per MinION flow cell.

A rule of thumb we use is that for cDNA, we get one R2C2 read of a cDNA molecule for 5 1D cDNA reads of the same length. We would argue that the LRGASP data isn’t quite comparable between R2C2 and ONT 1D cDNA because while the cDNA we sequenced with R2C2 had a median length of 1150nt (2500nt for the size-selected libraries), the ONT cDNA had a median length of 550nt. The two techniques are sampling entirely different distributions of molecules.

Also, for R2C2, the tradeoff between accuracy and throughput is very much tuneable by size-selecting RCA DNA before sequencing. For the cited LRGASP numbers, we really pushed accuracy. For our 2020 Genome Research paper (~2.5 million reads per MinION flowcell) we chose a more balanced approach as we did for our LRGASP Promethion data [ENCSR254DEM, ENCSR909KUO] which comes in at about 14 million reads per flow cell (with ~6kb raw reads). For our most recent paper where we use R2C2 to analyze Illumina libraries, we really pushed throughput (and took advantage of shorter inserts) to generate ~9 million reads of a MinION flow cell.

But obviously the fact remains that one can always generate more 1D ONT reads.

8. I'd argue that . . . . some of the isoforms can be validated by short-read sequencing confirming some of the novel reconstructions - i.e. that NIC transcripts have some reads which span that exon-exon boundaries. But this is fair enough, I think I'm more asking the % of transcripts which are "expected" which the authors discuss in response to Rev1 (comment 6)

Great news that our responses to Reviewer 1 covered this!

11. Is it in fact so that the primary limitation of length is in the early PCR steps? It seems to me that circularization for R2C2 might also have some dependency on the size of the molecule - have the authors investigated this? Later (comment 13) the authors assert that cDNA sequencing has a length bias presumably this is from the PCR as direct cDNA shown in Chen et al (bioRxiv 2021) has a different median read length than cDNA-PCR. It's not clear to me why the authors don't observe the same size bias - is this just because their splint anchors to the strand-switching oligo? Examining Figure S2 their modal read length seems to be down around the same range, though Table S1 reports a high median "raw" read length, that doesn't mean their consensus read length is that long?

As the reviewer points out, the length of cDNA we end up sequencing (<1000nt median) is relatively short and shorter than what we can get out of recent bulk experiments (like 1150nt in LRGASP) and easily sequence. So we have a strong assumption that the major limitation for molecule length is upstream of the R2C2 steps.

This is clearly not straight-forward and I am sorry we dropped the ball on explaining this better.

We addressed the length bias of gibson assembly based circularization in the original R2C2 paper and after that continuously in our lab. But it is not an easy thing to assess because so many different steps play into this bias.

Length bias associated with PCR based cDNA sequencing on ONT is composed of all the different biases of (1) RT, (2) PCR, (3) library preparation, and the (4) length bias of the sequencer itself.

R2C2 includes (1) and (2), adds the potential bias of the gibson based circularization, but removes or at least severely mitigates (3) and (4) because the length of the RCA product that we make libraries from and sequence is actually independent of the length of the cDNA we originally circularized.

An example of why this matters can be found in the LRGASP data where the PCR based cDNA kit by ONT generates a 550nt median length (similar to Chen et al.), while R2C2 (including PCR as well) on the same RNA yields about more than double that at 1150nt.

I hope this clears this up sufficiently.

12. The authors assert here that there are 5000 molecules produced per cell (Figure 1), but pretty clearly from Figure 1B R2C2 does not capture the number of UMIs (per cell rank) that Illumina does? This is further confusing as it seems that there were *fewer* genes detected per cell in Illumina than R2C2 (Figure S4). It isn't clear that this is saturation level sequencing even for the Illumina data - Zhang et al (PMID: 30472192) found that more sequencing allows more gene detection.

As we describe in the results section, we sequenced a total of 202,469,707 Illumina reads so about 70,000 reads per cell which we then collapsed to about 5,000 UMIs per cell. According to Zhang et al. and their Fig. S4, 70,000 reads falls on the part of the distribution where more reads produce a very small number of additional UMIs. So we do believe that we are at or very close to saturation level with our Illumina data.

However, the confusion is justified and stems entirely from a mistake we made on our end. Thank you very much for catching the issues with Figure S4! It seems like we plotted molecule numbers instead of gene numbers. We have no excuse for this error. We replaced the figure with a fixed version. The main point of the figure stands, i.e. gene numbers per cell are highly correlated between R2C2 and Illumina but now the figure is actually showing what it says it’s showing and is in line with the numbers in the manuscript. Further, it shows that for the same cell, R2C2 and Illumina detect very similar numbers of genes even though there are fewer R2C2 molecules. Although this is only a guess, I think this likely stems from R2C2 reads being much longer and therefore have a higher chance to overlap with a gene model.

Though I can accept the problem with mandalorian, I do think that a rarefication analysis _is_ within the scope, because it allows the reader to get a sense for _how deeply_ we need to sequence to detect isoforms. As nanopore sequencing (especially with R2C2, see comments above about Table 7) and PacBio sequencing for that matter both are relatively limited in numbers of reads/$ as compared to say Illumina (which admittedly only gives gene level information) - use of this method with experimental design requires a sense of the depth of sequencing needed. If not a rarefication analysis, then at least a discussion from other (perhaps bulk) data of guidelines for depth of sequencing needed per sample to achieve the numbers of cells needed.

We agree that this is an important piece of information for future long-read single-cell experiments. We are happy to expand on our discussion as suggested.

We added the following text to the discussion:

“In its current state, the 10X/R2C2 method we developed allowed us to generate isoform-level transcriptomes for Monocyte, B cell, and T cell populations. Because the different cell types were present in the analyzed PBMC sample at varying frequencies, Monocyte, B cell, and T cell transcriptomes contained reads derived from varying numbers of cells. For the B cell transcriptome, we used 625,334 reads derived from 179 B cells. Rarefaction analysis of full-length transcriptome sequencing in previous bulk experiments (Soneson et al. 2019) strongly suggests that this B cell transcriptome is almost certainly not saturated and sequencing more cells would result in a much more exhaustive isoform-level transcriptome. On the other hand, for the T cell transcriptome, we used 9,108,828 reads derived from 2,199 T cells. However, rarefaction analysis of full-length transcriptome sequencing of bulk RNA from a lymphoblastoid cell line (Workman et al. 2019) again suggests that even this sequencing depth might not yield a transcriptome at saturation.

Because the number of unique molecules generated per single cell is limited (~around 5000 in this study), increasing sequencing depth to reach transcriptome saturation in future single-cell studies will have to be accomplished by increasing the number of cells sequenced. The relative frequencies of specific cell types therefore will have to be taken into account when determining how many total cells to include in a single-cell experiment if the goal is to generate comprehensive transcriptomes for these specific cell types. The exact number of reads required to reach saturation will depend on cell type/state, the sequencing method, and the isoform-calling pipeline, but based on bulk studies will likely be above 10 million, which corresponds to more than 2000 cells.”
